# Intricate environment-modulated genetic networks control isoflavone accumulation in soybean seeds

**DOI:** 10.1186/1471-2229-10-105

**Published:** 2010-06-11

**Authors:** Juan J Gutierrez-Gonzalez, Xiaolei Wu, Jason D Gillman, Jeong-Dong Lee, Rui Zhong, Oliver Yu, Grover Shannon, Mark Ellersieck, Henry T Nguyen, David A Sleper

**Affiliations:** 1 Division of Plant Sciences and National Center for Soybean Biotechnology, University of Missouri, Columbia, MO 65211, USA; 2 USDA-ARS, 108 Waters Hall, University of Missouri, Columbia, MO 65211, USA; 3 Division of Plant Biosciences, Kyungpook National University, Daegu 702-701, Republic of Korea; 4 Donald Danforth Plant Science Center, 975 North Warson Road, Saint Louis, MO 63132, USA; 5 Department of Statistics, University of Missouri, 146 Middlebush Hall, Columbia, MO 65211 USA; 6 USDA-ARS Plant Science Research Unit and University of Minnesota, St Paul, Minnesota 55108, USA

## Abstract

**Background:**

Soybean (*Glycine max *[L] Merr.) seed isoflavones have long been considered a desirable trait to target in selection programs for their contribution to human health and plant defense systems. However, attempts to modify seed isoflavone contents have not always produced the expected results because their genetic basis is polygenic and complex. Undoubtedly, the extreme variability that seed isoflavones display over environments has obscured our understanding of the genetics involved.

**Results:**

In this study, a mapping population of RILs with three replicates was analyzed in four different environments (two locations over two years). We found a total of thirty-five main-effect genomic regions and many epistatic interactions controlling genistein, daidzein, glycitein and total isoflavone accumulation in seeds. The use of distinct environments permitted detection of a great number of environment-modulated and minor-effect QTL. Our findings suggest that isoflavone seed concentration is controlled by a complex network of multiple minor-effect loci interconnected by a dense epistatic map of interactions. The magnitude and significance of the effects of many of the nodes and connections in the network varied depending on the environmental conditions. In an attempt to unravel the genetic architecture underlying the traits studied, we searched on a genome-wide scale for genomic regions homologous to the most important identified isoflavone biosynthetic genes. We identified putative candidate genes for several of the main-effect and epistatic QTL and for QTL reported by other groups.

**Conclusions:**

To better understand the underlying genetics of isoflavone accumulation, we performed a large scale analysis to identify genomic regions associated with isoflavone concentrations. We not only identified a number of such regions, but also found that they can interact with one another and with the environment to form a complex adaptable network controlling seed isoflavone levels. We also found putative candidate genes in several regions and overall we advanced the knowledge of the genetics underlying isoflavone synthesis.

## Background

Considerable evidence has implicated isoflavones in the fitness of both humans and plants. A search of the literature reveals thousands of articles and subsequent reviews describing effects on human health associated with isoflavone consumption and possible molecular mechanisms of action (for recent reviews see [1-3]). Within the plant itself, isoflavones play a critical role in defense against fungal pathogens [4,5], and they also are required for the establishment and perdurability of nodules in rhizhobium-plant symbiotic associations [6,7]. Consequently, there is an increasing interest in altering the isoflavone content of soybean commercial varieties, which requires an understanding of the genetics governing their synthesis and accumulation. However, unmasking the genetics underlying isoflavone accumulation in seeds is challenging because: i) isoflavones display a broad range of variability over environments due to the many factors that affect their synthesis and accumulation [8,9]; ii) many QTL with small individual effects contribute in an additive manner [10-14]; iii) epistatic interactions are responsible for a great proportion of the observed phenotypic variance [14]; and iv) soybean features a complex genome which has undergone several whole genome duplication events [15]. As a result, tissue-differential expression or loss-of-expression or function of some of the resulting paralogs may have occurred.

Soybean seed isoflavone levels are subjected to a great oscillation because many biotic and abiotic factors influence their synthesis and accumulation [3,16-20]. For example, in a four-location field trial a cultivar was found to fluctuate from 460 to 1950 μg g^-1 ^in its isoflavone levels in seeds. Even within the same environment and year, an almost 3-fold variation was reported for a single cultivar [8]. Nevertheless, in spite of the environmental interactions, the control of isoflavone content in seeds is largely genetic [14,21-23], and numerous minor-effect QTL have been found to determine soybean isoflavone amounts. Unfortunately, many of them were identified solely based upon a particular environment, location and/or year. QTL that are stable over multiple environments are more useful in breeding programs because they might contribute to a consistent phenotype under changing conditions.

**Figure 1:**
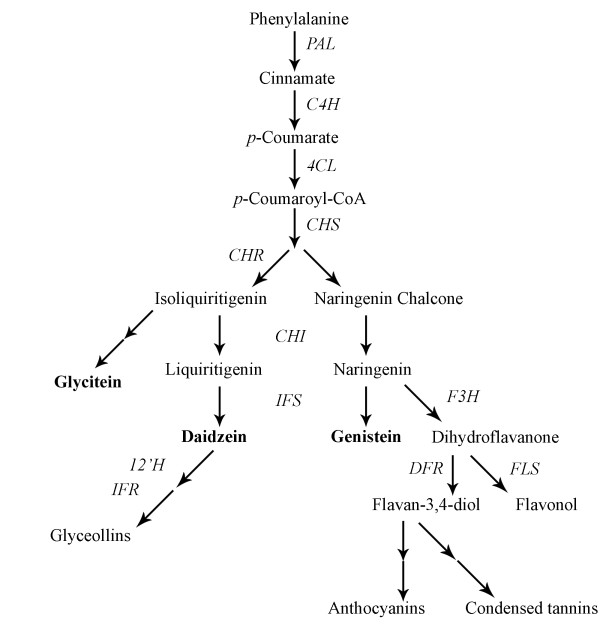
**Schematic representation of the phenylpropanoid pathway (adapted from **[56]). Phenylalanine ammonia-lyase (PAL), cinnamic acid 4-hydroxilase (C4H), 4-coumarate:CoA ligase (4CL), chalcone synthase (CHS), chalcone reductase (CHR), chalcone isomerase (CHI), isoflavone synthase (IFS), flavanone 3-hydroxylase (F3H), dihydroflavonol reductase (DFR), flavonol synthase (FLS), isoflavone hydroxylase (12'H), and isoflavone reductase (IFR).

A set of enzymes within the phenylpropanoid pathway are responsible for the biosynthesis of the three known soybean isoflavones: genistein, daidzein, and glycitein (Figure 1). Phenylalanine ammonia lyase (PAL), cinnamic acid 4-hydroxilase (C4H), and 4coumarate:CoA ligase (4CL) are the first enzymes in the pathway. They convert the amino acid phenylalanine into p-Coumaroyl-CoA. In the next step, the critical enzyme chalcone synthase (CHS) acts either alone or together with chalcone reductase (CHR) to form isoliquiritigenin and naringenin chalcone, respectively. Both are substrates of chalcone isomerase (CHI), which converts them into liquiritigenin and naringenin. These two compounds are the precursors of the soybean isoflavones daidzein and genistein, formed after the action of the key enzyme isoflavone synthase (IFS). The enzyme flavanone 3-hydroxylase (F3H) competes with IFS in the utilization of naringenin, in a branch of the pathway leading to the formation of flavonols, condensed tannins and anthocyanins. Daidzein can also act as a substrate, and after a series of reactions catalyzed by the enzymes isoflavone hydroxylase (I2'H) and isoflavone reductase (IFR), among others, leads to the synthesis of glyceollins. The biochemical steps leading to the third soybean isoflavone, glycitein, are not entirely known, although isoliquiritigenin is likely a precursor [24].

Cellular systems are intrinsically complex and their components often interact with one another in such an elaborate way that they may never be entirely understood. At the gene level, those interactions are known as epistasis. Under the quantitative genetics point of view, an epistatic interaction is revealed when a particular combination of alleles at distinct loci produces a different phenotype than when they are found apart. Thus, it is the particular combination of alleles that results in a change in the phenotype, i.e. the same allele can produce different phenotypes in a different genetic background. Epistatic effects have often been disregarded in mapping studies and left as a background distortion [25]. However, since additional knowledge has been garnered over the last several years, epistasis has passed from being considered the exception to the norm for many traits, and inter-gene interactions have been proven to account for a great proportion of the variability of complex traits, including isoflavones [14,26,27]. This 'extra' source of variation has been suggested to play an important role in biological processes such as heterosis, fitness and adaptation to different environments, and subsequently to evolution and speciation in natural populations [28-33]. Despite its importance, detecting epistasis genome-wide is not straightforward in experimental populations, and is certainly more difficult than for main-effect QTL. Statistical power to detect pairwise epistatic interactions is lower than for main QTL because tests of significance must be conducted for two intervals rather than just one, and consequently a higher critical threshold per test must be applied to overcome the problem of multiple tests [33]. This can be translated into small-effect interactions that would remain undetected unless a larger number of individuals are considered.

Interestingly, epistasis has been found to correlate with genomic complexity and the number of chromosomes [34,35]. The genome of soybean is known to be quite complex [15], owing to two presumed recent whole genome duplications (recently reviewed in [36]). As a result, a single-copy gene in Arabidopsis can typically be expected to be present as two or four homologous genes in soybean [37]. Gene duplication is frequently associated with either tissue-specific differential expression (a process termed subfunctionalization), the acquisition of a new mode of action (neofunctionalization), or loss of expression or function of one or more copies (pseudogene formation) [38]. Naturally, phenylpropanoid genes are not oblivious to this complexity and attempts to genetically modify the pathway have sometimes led to unexpected results. For example, over-expression of key enzymes such PAL, CHS, or IFS, either independently or combined, have failed to enhance isoflavone accumulation in seeds [39,40].

Over fifty loci have been reported in the few QTL mapping studies conducted on isoflavones [10-14]. However, a great majority has not been confirmed by using different parental lines, locations, or years. Attempts to validate the QTL have failed in part due to the nature of isoflavone QTL, with predominant minor main-effect, for which uncovering the experimental design needs to be optimized with, for instance, larger number of individuals and replications, enough and evenly distributed markers, and an adequate mapping methodology. We previously used the Essex × PI437654 cross to study the genetic control of seed isoflavone content [14]. Herein, we first added data from one additional year and two locations, with three replications per location, to perform a comprehensive mapping analysis by a mixed linear model approach aimed at discovering not only the most stable main and epistatic QTL over environments, but also the QTL environment-modulated. Mixed linear models have been proposed as an efficient approach to assess environmental effects [41]. Second, we conducted an exhaustive search for candidate genes with homology to important genes for isoflavone synthesis. Genes were placed onto the genetic map together with the QTL identified in an attempt to validate QTLs by finding candidate genes, and thus, to begin to decipher the genetic network underling soybean isoflavone seed content.

## Results

### Genetic and phenotypic variation within mapping population

**Figure 2:**
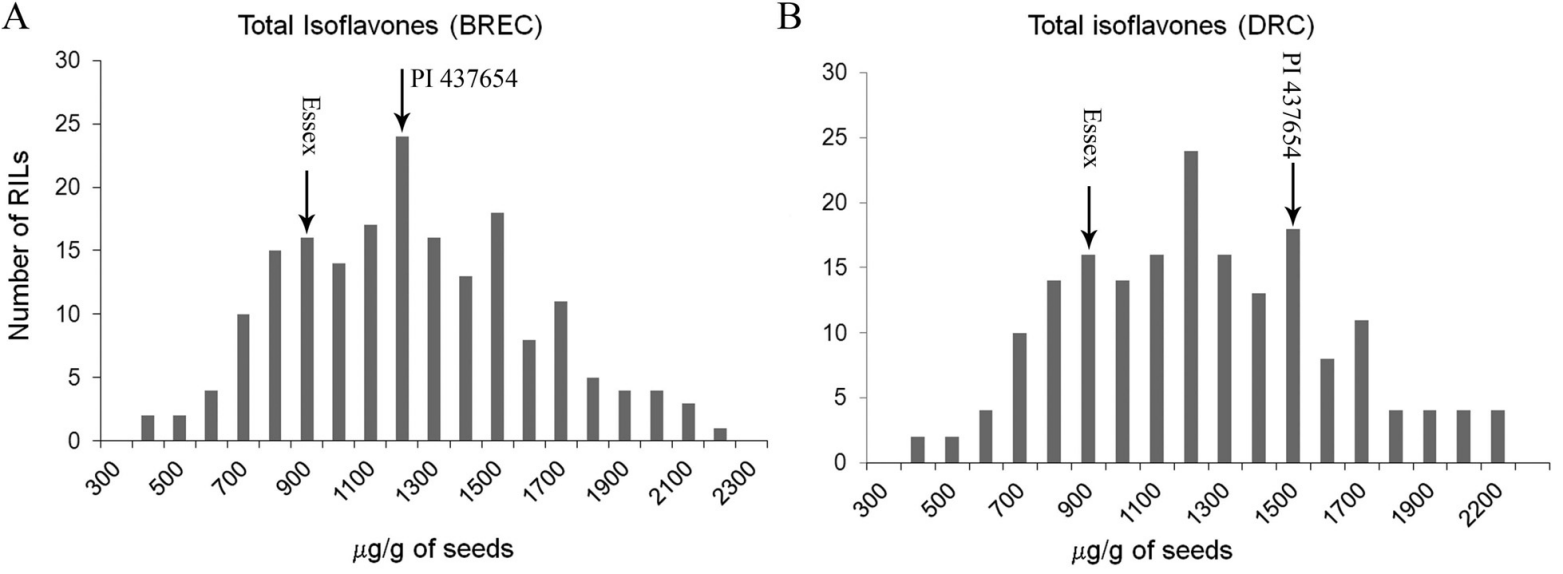
**Distribution of averaged total isoflavones in three replications of Essex × PI 437654 RILs growing in two field locations, BREC (A) and DRC (B) in year 2007**. See supplemental material for individual isoflavones (year 2006 data detailed in [14]). Arrows indicate the position of the two parental lines. Horizontal axis shows each particular isoflavone seed content in μg/g of seeds.

Analysis of variance (ANOVA) and heritabilities in the broad sense (H^2^) were calculated over the combined four environments as they are good indicators of the origin of the variation within segregating mapping populations (Table 1). Results confirmed that the accumulation of isoflavones in soybean seeds is highly influenced by genetic (G), environmental (E), and G×E interaction effects (P < 0.0001). Furthermore, the high heritability values (0.76-0.92) suggested that, despite environmental influence, a high proportion of the phenotypic variation is determined by genes. In addition, because H^2 ^estimated on RILs encompasses only additive (A) and additive-by-additive (AA) epistatic genetic variances, the involved genes could be acting alone or interacting. Frequency distribution and normal-distribution parameters of isoflavone seed content between locations and years clearly indicated quantitative inheritance of these traits (Table 1, Figure 2, and Additional file 1). Although parental lines did not greatly differ in genistein, daidzein and total isoflavones in some environments, considerable transgressive segregation was found, which indicated that both parents bear positive-effect alleles for isoflavone synthesis, and ultimately suggested that an elevated number of QTL might be segregating and likely to be detected in the mapping analysis.

**Table 1:** ANOVA, Effect Mean Squares, and heritability over environments of the mapping population.

				**Effect Mean Squares**^c^						
							
Trait	**Mean ± SD**^a^	Range	**CV**^b^**(%)**	V(G)	V(E)	V(GE)	V(ε)	**H**^2d^	Skewness	Kurtosis
Genistein	730.4 ± 288.3	82--1980	39.47	425223.1	14106588.3	56107.6	17610.1	0.88	0.409	-0.078
Daidzein	417.0 ± 210.1	33--1477	50.37	314677.8	2505067.7	28451.8	8586.1	0.92	0.798	0.990
Glycitein	126.8 ± 61.8	0--342	48.75	11792.1	1057117.8	3582.1	524.9	0.76	0.460	-0.580
Total Isoflav.	1272.7 ± 485.0	186--3008	38.11	1522309.0	19163038.5	163081.8	49308.0	0.90	0.350	-0.186

^a^Mean ± Standard deviation (SD) and range units in μg of isoflavones per gram of seeds.

^b^Coefficient of variation within the segregating mapping population over environments.

^c^Variance of genetic V(G), environmental V(E), genotype by environment interaction V(GE), and residual V(ε) effects.

^d^Heritability of the traits in the broad sense over environments.

### QTL Mapping shows that numerous minor-effect loci control isoflavone amounts

**Figure 3:**
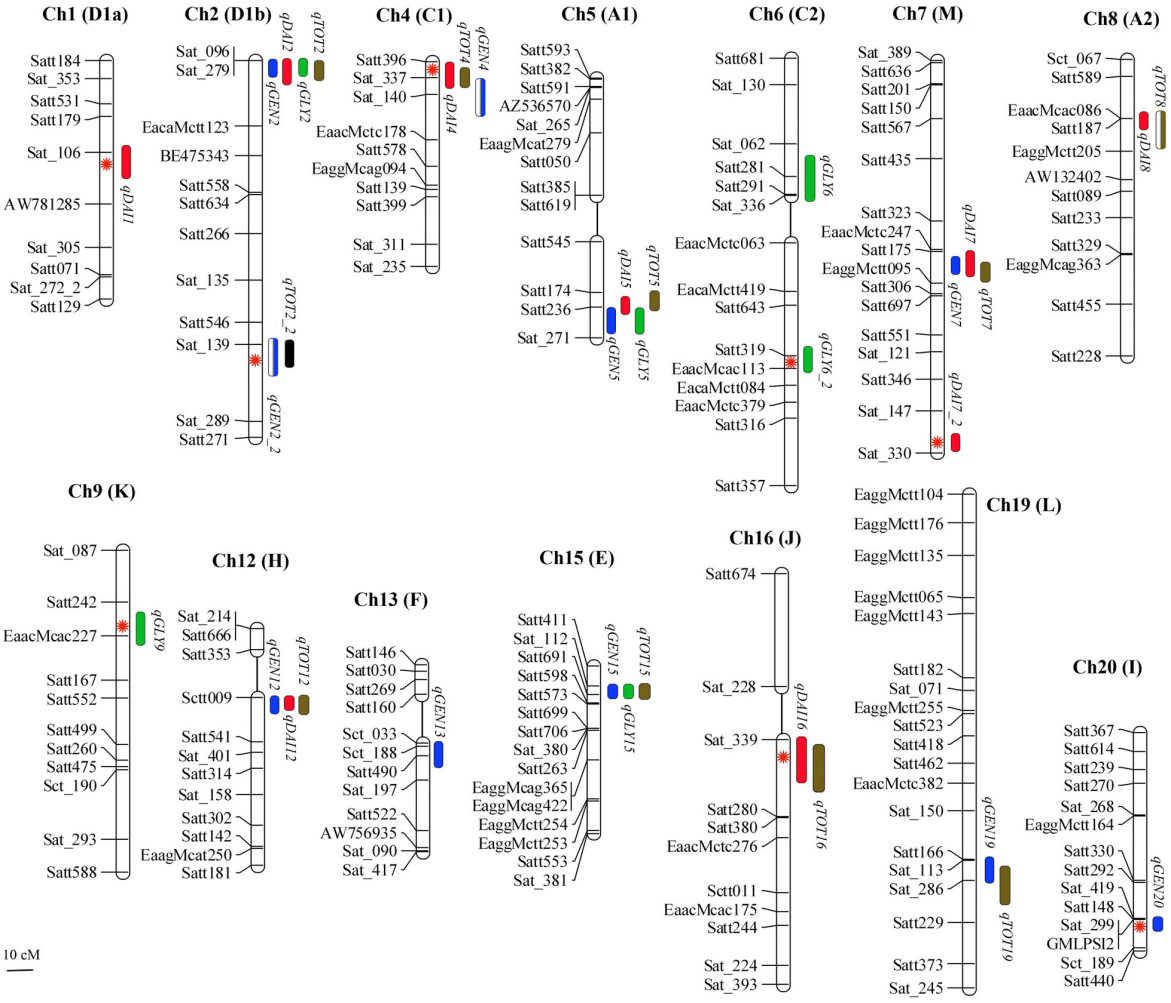
**Linkage group map summarizing QTL locations detected for distinct isoflavone species**. Blue ovals indicate additive main-effect loci associated with genistein, red ovals indicate loci associated with daidzein, green ovals indicate loci associated with glycitein and brown ovals indicate loci associated with total isoflavones. Hatched ovals indicate particular loci discovered only in one location, when data was run separately by locations: hatched-brown indicate total isoflavone content at BREC in year 2006 and hatched-blue genistein at BREC in 2007. Flanking and other key markers used for linkage analysis are depicted at the left side of the linkage group. The name of the QTL, shown aside each oval, is a composite of the influenced trait: genistein (*GEN*), daidzein (*DAI*), glycitein (*GLY*) and total of isoflavones (*TOT*), followed by the chromosome number. Red asterisks show newly discovered QTLs while no asterisks indicate confirmation QTL.

Developing RILs may be time-consuming but allows for testing the same genetic background under different conditions. Seed isoflavone content is highly affected by the environment (Table 1). Hence, it is not surprising that very distinct sets of QTL data have been reported when the same mapping populations were independently analyzed under different conditions [12,14]. In this study, data from four different environments were used to perform an integrated analysis by a mixed linear model. A total of 2460 (205 RILs × 3 replications × 4 locations) individual observations were used to run the mapping software. Running data from different locations and individual replicates together under a unique model has been proven to be a powerful tool for discovering minor-effect QTL in complex traits because of the contribution to a reduced experimental error associated with increased observations [14,41], and might also allow the identification of the most stable QTL over the pooled locations. As a result, ten additive main genomic regions were found to influence genistein accumulation in seeds (Table 2 and Figure 3). A major QTL was found on chromosome 5 (Gm05, following the new nomenclature for soybean chromosomes) named *qGEN5*, with an estimated heritability of the additive effect, h^2^(a), of 5.5%. Other identified important regions were in Gm02, Gm15, Gm13, Gm19 and Gm07, which were named *qGEN2*, *qGEN15*, *qGEN13*, *qGEN19*, and *qGEN7*, respectively. All were previously reported [14]. Importantly, two newly identified loci were found in Gm12 and Gm20, *qGEN12 *and *qGEN20*. Among them, *qGEN5*, *qGEN2*, and *qGEN19*, also displayed an additive by environment (A×E) interaction effect, reflecting differences in accumulation over locations (Additional file 2). We also used each environment alone as input data for the mapping software. As a result, two other regions were found to explain genistein accumulation: *qGEN4 *and *qGEN2_2 *(at BREC in 2007). For daidzein, nine different regions were exerting influence on its accumulation (Table 2 and Figure 3). Two key loci were found in Gm05 and Gm12, *qDAI5 *and *qDAI12*. Other influencing loci were mapped in Gm08, Gm04, Gm01, Gm02, and Gm16: *qDAI8*, *qDAI4*, *qDAI1*, *qDAI2*, *qDAI16*, respectively, and two in Gm17, *qDAI7 *and *qDAI7_2*. Apart from their additive effect, *qDAI2*, *qDAI12*, and *qDAI17_2 *also showed a significant A×E interaction effect. Glycitein accumulation was found to be influenced by a total of six different genomic regions (Table 3 and Figure 3). Two of them, *qGLY15 *and *qGLY6*, explained a greater percentage of the variation than any other QTL. Newly found glycitein loci were detected in Gm05, Gm06, Gm02, and Gm09 (*qGLY5*, *qGLY6*, *qGLY2*, and *qGLY9*). Reflecting the variability of this particular isoflavone over environments, five out of six loci had an A×E interaction effect. In the latter instance, total isoflavone content was mapped as the sum of the previous three, resulting in the discovery of ten genomic regions that influenced its content in seeds. The most influencing locus was again placed in Gm05 (*qTOT5*). This was previously reported, as well as the other five (*qTOT2*, *qTOT15*, *qTOT12*, *qTOT19*, and *qTOT7*). Newly discovered regions were found in Gm04 (*qTOT4*), Gm02 (*qTOT2_2*), and Gm16 (*qTOT16*). Another QTL (*qTOT8*) was detected only at BREC in 2006. Five of these QTLs also bared a component of A×E interaction effect. The total percentage of phenotypic variance accounted for additive QTLs were 24.5, 24.3, 7.7, and 25.9% for genistein, daidzein, glycitein and total isoflavones, respectively.

**Table 2:** Additive QTLs for genistein and daidzein accumulation in soybean seeds of Essex × PI 437654.

Name^a^	Interval	IC^b^	A ± SE^c^	F-value^d^	P-Value	h^2^(a)^e^
Genistein						
						
qGEN5*	SATT236-SAT_271	27.4-34.4	82.1 ± 5.2	35.2	0.00000	5.5
qGEN2*	SAT_279-EACAMCTT123	0.0-6.0	54 ± 4.3	34.7	0.00000	2.0
qGEN15*	SAT_112-SATT691	8.0-11.0	58.9 ± 4.6	24.8	0.00000	2.3
qGEN13*	SATT490-SAT_197	3.4-11.0	25.3 ± 4.4	13.1	0.00000	1.3
qGEN12	SCTT009-SATT541	0.0-6.0	41.9 ± 4.3	11.9	0.00000	1.2
qGEN20	GMLPSI2-SCT_189	72.5-74.5	-32.5 ± 4.1	8.1	0.00000	0.4
qGEN19*	SAT_113-SAT_286	141.4-149.8	-47.5 ± 4.5	13.3	0.00000	1.9
qGEN7*	SATT175-EAGGMCTT095	76.1-83.1	42.9 ± 4.9	14.7	0.00000	2.3
qGEN4	SAT_337-SAT_140	6.2-17.6	-47.5 ± 7.2	19.8(17.3)	0.00000	3.3
qGEN2_2	SAT_139-SAT_289	107.6-126.2	-44.8 ± 9.0	27.2(17.3)	0.00001	5.3
Daidzein						
						
qDAI5*	SATT174-SATT236	21.5-28.4	50.3 ± 3.2	32.8	0.00000	8.3
qDAI8*	SATT187-EAGGMCTT205	23.0-25.1	-27.9 ± 2.9	17.5	0.00000	2.2
qDAI4	SATT396-SAT_337	0.0-6.0	-27.5 ± 3.2	15.7	0.00000	1.2
qDAI1	SAT_106-AW781285	34.7-42.6	20.9 ± 3.1	14.2	0.00000	1.2
qDAI2*	SAT_279-EACAMCTT123	0.0-8.0	27.5 ± 3.2	20.4	0.00000	1.4
qDAI12*	SCTT009-SATT541	0.0-3.0	63.6 ± 3.1	25.2	0.00000	4.3
qDAI16	SAT_339-SATT280	0.0-15.0	-58.8 ± 3.9	15.5	0.00000	2.3
qDAI7*	SATT175-EAGGMCTT095	75.1-83.1	55.7 ± 3.5	13.6	0.00000	2.4
qDAI7_2	SAT_147-SAT_330	145.7-151.7	-38.5 ± 3.1	9.2	0.00000	1.0

^a^Name given to a particular QTL, *GEN *and *DAI *for genistein and daidzein content, respectively, followed by the chromosome number and a number when more than one in the same chromosome. Asterisks highlight QTL reported in our previous study [14]. ^b^Interval of confidence in centiMorgans with respect to the first marker in the LG. ^c^Main additive effect in μg/g plus/minus standard error. Mean effect of substituting both Essex alleles by PI437654 alleles. Thus, positive values indicate that the PI437654 allele increase the phenotypic value. ^d^F-values of significance of each QTL. Threshold F-values were 7.4 and 7.5 for genistein and daidzein, respectively, for the 4-environmentcombined analysis. Between brackets is the threshold F-value for QTLs identified at a particular environment. P-values represent the significance of each effect. ^e^h^2^(a) is the heritability of the additive effect or percentage of variation (%) that is explained by the additive component of the QTL.

**Table 3:** Additive QTLs for glycitein and total isoflavone accumulation in soybean seeds of Essex × PI 437654.

Name^a^	Interval	IC^b^	A ± SE^c^	F-value^d^	P-Value	h^2^(a)^e^
Glycitein						
						
qGLY5	SATT236-SAT_271	26.4-35.4	7.1 ± 1.1	18.2	0.00000	1.4
qGLY6*	SATT281-SATT291	40.0-52.7	-7.1 ± 0.9	9.2	0.00000	1.4
qGLY6_2	SATT319-EAACMCAC113	41.2-48.8	9.2 ± 0.9	8.3	0.00000	1.0
qGLY2	SAT_279-EACAMCTT123	0.0-5.0	5.9 ± 0.8	13.7	0.00000	0.9
qGLY15*	SAT_112-SATT691	8.0-11.0	10.7 ± 1	19.9	0.00000	2.1
qGLY9	SATT242-EAACMCAC227	25.1-36.3	6.4 ± 0.9	10.0	0.00000	0.9
Total						
						
qTOT5*	SATT174-SATT236	20.5-25.4	161.7 ± 8.3	36.7	0.00000	7.0
qTOT4	SAT_337-SAT_140	3.0-9.2	-104.1 ± 7.8	9.8	0.00000	1.1
qTOT2*	SAT_279-EACAMCTT123	0.0-7.0	87.4 ± 8.1	34.7	0.00000	2.1
qTOT2_2	SAT_139-SAT_289	107.6-118.2	-63.2 ± 7.7	11.6	0.00000	1.9
qTOT15*	SAT_112-SATT691	8.0-11.0	52.9 ± 8.5	15.0	0.00000	1.7
qTOT12*	SCTT009-SATT541	0.0-4.0	95.2 ± 7.8	20.5	0.00000	3.4
qTOT16	SAT_339-SATT280	3.0-19.0	-125.8 ± 11.2	9.2	0.00000	1.3
qTOT19*	SAT_286-SATT229	144.8-159.9	-69.9 ± 8.8	14.3	0.00000	1.4
qTOT7*	SATT175-EAGGMCTT095	76.1-85.1	115.8 ± 9	11.0	0.00000	3.1
qTOT8*	SATT187-EAGGMCTT205	23.0-29.1	-50.5 ± 14.6	19.3 (16.2)	0.000554	3.0

^a^Name given to a particular QTL, *GLY *and *TOT *for glycitein and total isoflavone content, respectively, followed by the chromosome number and a number when more than one in the same chromosome. Asterisks highlight QTL reported in our previous study [14]. ^b^Interval of confidence in centiMorgans with respect to the first marker in the LG. ^c^Main additive effect in μg/g plus/minus standard error. Mean effect of substituting both Essex alleles by PI437654 alleles. Thus, positive values indicate that the PI437654 allele increase the phenotypic value^. d^F-values of significance of each QTL. Threshold F-values were 7.2 and 8.0 for glycitein and total isoflavones, respectively, for the 4-environmentcombined analysis. Between brackets is the threshold F-value for QTLs identified at a particular environment. P-values represent the significance of each effect. ^e^h^2^(a) is the heritability of the additive effect or percentage of variation (%) that is explained by the additive component of the QTL.

### Epistasis is decisive in determining seed isoflavone amounts

Accumulated evidence suggests that gene-gene interactions may be accountable for a great part of the phenotypic variation observed in complex traits. Particularly, additive × additive (AA) epistatic interactions have been proven to be responsible for a great part of isoflavone seed amounts in certain environments, even more than main QTL [14]. Despite its interest, mapping epistasis in complex traits is a daunting task because of the great number of pair-wise combinations implicated and their small individual effects. Analogous to the additive main-effect QTL, performing a combined analysis with replicated observations and over different environments allows not only recovery of a great number of small-effect AA epistasis, but also the more stable-over-environment. A total of fourteen, twenty one, seven, and eight AA epistatic interactions were found to influence genistein, daidzein, glycitein and total isoflavones accumulation, respectively (Additional file 3). In addition to the AA epistatic main-effect, some of the interactions also possessed an AA×E interaction effect (Additional file 4). The importance of epistasis was most revealed for daidzein, for which the percentage of the variation explained for by the sum of epistatic interactions was 22.7%. For genistein, glycitein and total isoflavones this percentage was 10.7, 4.7, and 12.0, respectively. Importantly, the maximum contribution by any particular interaction was only 3.6%. All chromosomes had at least one epistatic connection. Overall, our results suggested that epistasis is a major determinant of phenotypic variance for isoflavone seed contents, and that many small individual interactions contribute to the total effect.

### Isoflavone synthesizing genes as candidate genes for additive QTL

**Figure 4:**
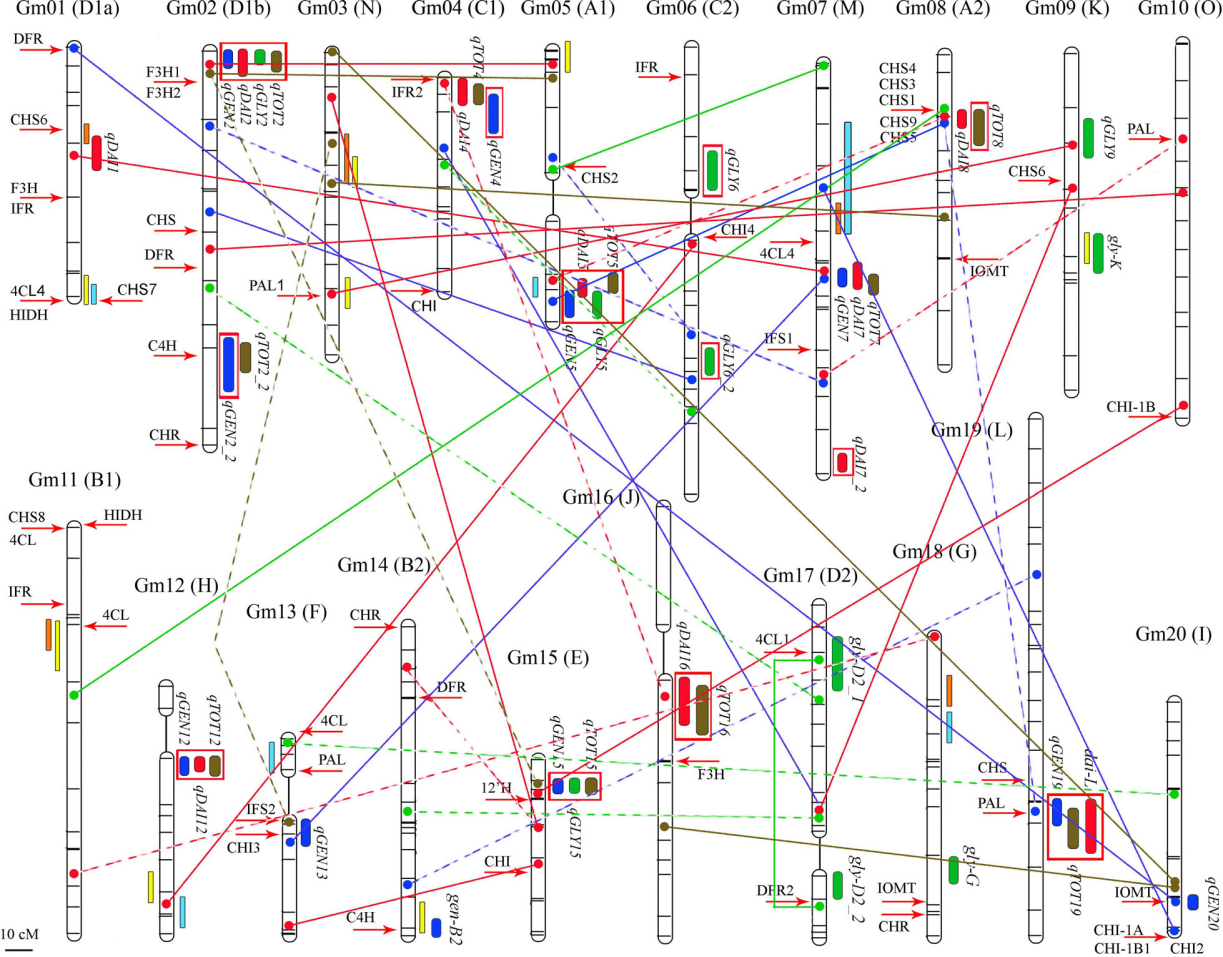
**Environment-modulated network of additive main-effect and interacting QTL controls isoflavone accumulation**. Blue ovals indicate additive main-effect loci associated with genistein, red ovals indicate loci associated with daidzein, green ovals indicate loci associated with glycitein and brown ovals indicate loci associated with total isoflavones. The name of the QTL, shown aside each oval, is a composite of the influenced trait: genistein (*GEN*), daidzein (*DAI*), glycitein (*GLY*) and total of isoflavones (*TOT*), followed by the chromosome number. Red squares surround the loci with effect-by-environment interaction, i.a. QTL with a significant additive component but also significantly different at each environment. In an effort to make the map more informative QTL reported for ours and other groups: yellow squares [10], light-orange [11], and light-blue squares [12]. Lines indicate en epistatic interaction between the interconnected genomic regions, maintaining the same color codes than for additive QTLs: blue for genistein, red for daidzein, green for glycitein, and brown for total isoflavone content. Dotted lines reflect epistasis with effect-by-environment interaction component. Genomic locations of candidate genes for isoflavone synthesis are located on the chromosomes by a red arrow and their abbreviated name.

The recent release of the whole genome sequence Glyma1 assembly for Williams 82 [15] (accessible at http://www.phytozome.net/soybean) provides a powerful tool with which to interrogate QTL data. Previously reported genes for isoflavone biosynthesis [3] were used in BLAST searches against the whole genome sequence to identify homologous regions in the genome with assigned or putative functions (Additional file 5). All twenty soybean chromosomes had regions sharing a high percentage of homology with genes of known function in the phenylpropanoid pathway (Figure 4). In an effort to add more information onto the network map, we also included other reliable QTL reported earlier by our and other groups [10-12,14]. In total, out of the twenty soybean chromosomes that form the soybean genome, nineteen were found bearing at least one additive QTL accounting for seed isoflavone accumulation, reflecting the wide-spread distribution of isoflavone influencing loci. In addition, to make the genetic map as informative as possible, all homologous sequences found during the blast search were also placed on the map at their approximate positions, including the ones with only putative function or potential pseudogenes. Candidate genes were considered those falling in the interval of confidence (IC) of a main-effect QTL, assuming the limits of the mapping resolution.

Accordingly, several matches were found between QTL and biosynthetic genes. In Gm01, *qDAI1 *and another locus reported somewhere else for glycitein synthesis [11] overlapped a copy of chalcone synthase (*CHS6*). Another region at one of the ends of the chromosome was previously reported to account for glycitein seed content [10,12] and embraced three phenylpropanoid genes (*CHS7*, *4CL4*, and *HIDH*). Nearby the end of Gm02, two *F3H* copies matched the position of four QTLs (*qGEN2*, *qDAI2*, *qGLY2*, and *qTOT2*). In the same chromosome, two other QTL were found to explain genistein and total isoflavones (*qTOT_2 *and *qGEN2*). A *C4 H *gene is centered in their IC. Another *C4 H *copy on Gm14 is found within the interval of the QTL *gen-B2*, and also of two other QTL for genistein and daidzein previously reported [10]. In Gm03 a glycitein QTL found by the same group [10,11] coincides with a copy of another important gene, *PAL1*. At the beginning of Gm04, three QTL (*qGEN4*, *qDAI4*, and *qTOT4*) were in the same region in which an isoflavone reductase copy (*IFR2*) was mapped. Several genomic regions in Gm07 account for the synthesis of distinct isoflavones (*qGEN7*, *qDAI7*, *qTOT7*, and *qDAI7_2*). However, despite the observation that critical isoflavone genes (*IFS1 *and *4CL4*) are present in this chromosome, those QTLs remain without a clear candidate. An extensive area of approximately 50 cM was shown to influence genistein, daidzein, glycitein and total isoflavone accumulation [12]. This region was apparently reduced to ~15 cM by another group [11], because the latter is included in the former and both accounted for the same isoflavone compounds. The *4CL4 *gene is less than 5 cM apart from this region and about 10 cM from *qGEN7*, *qDAI7*, and *qTOT7*. Numerous QTL reported for this chromosome suggests the presence of a large number of polymorphisms associated with isoflavone synthesis. In Gm11, a region identified to affect glycitein content [10,11] was found to overlap a *4CL *homolog. A chalcone isomerase (*CHI3*) and isoflavone synthase (*IFS2*) genes were located in the region identified by *qGEN13*, in Gm13. In this chromosome, another QTL was reported for glycitein [12], with a *PAL *and *4CL *copies lying nearby the QTL IC. In Gm14, one of the two *C4 H *homologous copies is within the genomic area delimited by *gen-B2 *[14] and by two other QTL for genistein and daidzein [10]. The only known copy of the isoflavone hydroxylase (*I2'H*) gene is in the region delimited by the IC of *qGEN15*, *qGLY15*, and *qTOT15*, in Gm15. Two glycitein loci were previously reported by our group in Gm17 (*gly-D2_1 *and *gly-D2_2*), had a copy of *4CL1 *and *DFR2 *as candidate genes, respectively. Another QTL for glycitein in Gm18 (*gly-G*) is nearby a *IOMT *and *CHR *copies, although not overlapping. A phenylalanine ammonia-lyase copy (*PAL*) located on Gm19 is clearly a good candidate for *qGEN19*, *qTOT19*, and *dai-L*. Finally, in Gm20, a copy of the *IOMT *gene is within the region demarcated by *qGEN20*. In addition, copies of *CHI-1A*, *CHI-1B1*, and *CHI2 *are present in close proximity (<10 cM).

## Discussion

### Genetic control of isoflavone seed content

The phenotype of complex traits is the result of diverse genetic and environmental factors, many of which have been found to interact with one another. Isoflavone content in soybean seeds is highly variable among lines, locations, and within cultivars. The origin of such variability was found in genetic (G), environmental (E), and G×E interaction factors. This grade of phenotypic unpredictability inherent to the environment has long hampered the use of molecular breeding technologies, such as marker-assisted selection (MAS), to develop appropriate isoflavone lines. Besides, the need for pyramiding of numerous genes, most with very small effects and the inconsistency in estimation of those effects may make MAS quite difficult. Different MAS approaches have been successfully attempted targeting traits governed by many minor-effect QTL [42]. However, these approaches rely on finding a close marker-trait association relatively independent from the environment. Despite the environmental interference, heritability in the broad-sense was found high for all traits, which in a population of RILs indicates that the observed phenotypic variability is largely under the genetic control of additive loci, either by itself or by AA epistasis. Supporting the broad range of variation observed for isoflavones, several loci also have an E and a QTL×E interaction effect (A×E or AA×E) by themselves (Figure 4 and Additional files 2, 3 and 4).

Our multi-environment approach allowed detection of thirty-five main-effects QTL. Nearly 92% of them individually account for less than 5% of the phenotypic variability, suggesting that isoflavone seed concentrations are governed by numerous minor-effect QTLs. Successfully detecting QTL and interactions of such small effects could have been accomplished through including data points of various environments and replications as independent entities under the same mapping algorithm. Certainly, the presence of false positives cannot be ruled out but, likely, the many individual observations (2640) resulted in increased power and precision by reducing the experimental error [41]. Many of these main-effect QTL were also interacting, implying that they might have different absolute effects in different genetic backgrounds and environments. However, most of the interactions occurred between loci that bear no additive effects. Our results indicate that epistatic network of interactions for isoflavones is largely changed when plants are grown in different environments and may be one of the main causes of their phenotypic variability over locations and years. Corroborating this observation, approximately one-third of the epistatic interactions detected in this analysis also showed interaction with the environment (QTL×E). The same proportion of QTL×E interactions was found for different traits in a study in maize [43]. Recently, it has been shown that an epistatic interaction can have a positive or negative effect upon plant fitness depending on the environment by regulating the salicylic acid stress signaling pathway [44]. We hypothesize that the whole network of main-effect and epistatic QTL changes to confront and adapt to external biotic or abiotic stimuli. This is in agreement with the role of isoflavones in defense mechanisms against damaging agents such as pathogens, UV radiation and cold stress [5,16,17,45-47]. However, the specific reason why isoflavones are directed towards accumulation in seeds remains elusive.

Some observations suggest that we might currently perceive only the tip of the iceberg and that the genetic network controlling isoflavone synthesis is likely to be even more intricate than previously thought. First, the threshold applied to QTL detection must be very strict to deal with the multi-test issue and keep the rate of false positives low. For such complex traits likely imply that many minor-effect QTL would remain undiscovered, especially in sample mapping populations. This issue is even more critical for epistatic QTL because two intervals must be tested. For instance, taking into account both main and epistatic QTL, our model was only able to explain 37.9% of the variance (additive:25.8 + epistatic:12.1), 49.1% (25.4+23.7), 16.7% (10.2+6.5), and 40% (27.5+12.5) for genistein, daidzein, glycitein and total isoflavones, respectively. The high percentage of remaining unassigned variance is likely due to minor-effect loci and epistasis that did not reach the threshold level of detection, further suggesting that numerous minor-effect loci are involved in isoflavone synthesis. Underestimating the number of QTL causes overestimation of the genetic effects of the ones identified because of what is known as the Beavis effect [48,49]. Therefore, special care must be taken when considering the reliability of the parameter estimates. Second, only polymorphic loci will segregate in the progeny and consequently be detected. Important but monomorphic loci may remain undiscovered. This is especially crucial for epistatic QTL as both interacting loci must be polymorphic, as otherwise they will not create phenotypic variance. Third, a population of RILs may underestimate the total epistasis if dominant interactions exist [50]. Effectively, due to the structure of the mapping population, it is not possible to map QTL bearing dominant (D) main or epistatic effects (DD, AD or DA). However, due to the smaller number of genotypic classes, a RIL population increases the statistical power to detect the remaining AA component, which is the more useful for MAS because it is heritable [51]. Moreover, the self-pollinating nature of soybean, with usually less than 1% cross-pollination [52], does not suggest those effects to be important in natural populations. Fourth, third- or higher-order epistatic interactions are not reflected on the mapping analysis but could exist and be an important component for complex traits such as isoflavones [53,54]. As a last observation, despite the marker-dense linkage map and the threshold values considered for QTL detection, the presence of spurious QTL cannot be completely discarded due to the limited number of individuals sampled. Nevertheless, large sample size in experimental populations is not only economically unfeasible but also does not guarantee detecting all possible minor-effect QTL [55].

### Complex networks of interacting genes govern isoflavone content in soybean seeds

The soybean genome is believed to have undergone at least two independent duplications from a diploid ancestor to render the actual polyploidy [15,37]. This degree of duplication is also reflected in the phenylpropanoid genes and increases the difficulty in performing gene-function association analysis because polyploidization may bring about gain-of-function, loss-of-function or neo-functionalization of certain copies [38]. It is also common to find tissue-specificity for some gene copies. For example, *CHS *has nine paralogues distributed along seven chromosomes, out of which *CHS5 *and *CHS6 *were not found in seeds at detectable levels [56]. With regards to *CHS6*, it has been suggested that it is present as a single copy in Gm09 [57,58]. However, our BLAST searches revealed a second matching locus in Gm01 (Glyma01g22880.1), which features only a few SNPs when compared to the NCBI entry for *CHS6 *(Additional files 6, 7, 8 and 9).

A separate discussion of the *CHS *genes present on Gm08 is merited. According to BAC sequencing, as many as 12 *CHS*-related genes are thought to be present on Gm08, organized in two clustered regions [58]. One such region is composed of two perfect inverted repeated regions each containing *CHS1*, *CHS3*, *CHS4 *genes. This region is commonly referred to as the *I *locus, and although the specific silencing RNA is unclear, appears to act through siRNA-mediated gene silencing to inhibit seed coat pigmentation by specific degradation of seed coat specific *CHS7 *and *CHS8 *transcripts [58-60]. The *CHS6*, *CHS7 *and *CHS8 *genes are not apparently present in clusters on Gm08 but rather in single copy regions on other chromosomes [58]. In addition, at least four additional *CHS *genes (*CHS5, CHS3, CHS1*, and *CHS9*) are thought to be present in another *CHS *cluster on Gm08, for a total of 12 *CHS *genes on Gm08. The current Glyma 1.01 assembly of Gm08 contains only 7 *CHS*-corresponding regions (6 *CHS *gene models, and an additional matching region which does not have an annotated gene model, Additional file 5) as determined by BLAST searches using *CHS *sequence as queries http://www.phytozome.net/soybean. It seems likely that a miss-assembly has occurred in this region and the number of *CHS *genes present on Gm08 remains to be resolved. It is not currently clear to what extent other similar regions with gene repeats may have impacted on the assembly of the whole genome shotgun sequence. Furthermore, as many as ten regions were found sharing homology to the *IFR *gene. Although several of them may be pseudogenes, genes inactivated by the accumulation of deleterious mutations, it may be quite difficult to ascertain in which tissues, if any, these genes are expressed if they share a high percentage of homology. In addition, we currently have only a single sequenced genome (Williams 82) for reference. It is highly likely that other cultivars will have differing numbers and dispositions of phenylpropanoid genes. In particular, large scale rearrangements and deletions have been noted for the *CHS *gene clusters present on Gm08 [61].

The network of genes and interactions appears to have several interconnected neuralgic centers. The *CHS *gene cluster in Gm08 emerges as key node controlling the synthesis and accumulation of all individual isoflavones. The cluster rests within the IC of two important QTL for daidzein and total isoflavone content. In addition, it has connections with two other influential QTL and candidate genes: in Gm05, and Gm19(*PAL*). Importantly, all three isoflavones have at least one epistatic line converging in the *CHS *cluster. One of those pair wise interactions connected the cluster with the focal locus at the end of Gm05, and explained both genistein and daidzein, which is a rare phenomenon and further validated the interaction. This locus itself accounted for genistein, daidzein, glycitein, and total isoflavones (*qGEN5*, *qDAI5*, *qGLY5 *and *qTOT5*), and it is by far the most principal of all QTL reported herein in terms of explained percentage of variance and additive value. However, for this QTL of largest effect, no known biosynthetic gene was found to be located within this region. Perhaps a heretofore unknown isoflavone biosynthetic gene is within this region. Alternately, a *trans*-acting factor affecting the expression or activity of isoflavone biosynthetic genes may be present (transcription factor, ubiquitin-related protein, etc). Consistent with this hypothesis, the epistatic interactions that this locus has with another central locus (Gm08/*CHS *cluster) reinforces the hypothesis of an action through *trans*-regulatory control. Whether this region contains such a factor remains for future fine-mapping work. Strengthening its central role, the *CHS *cluster is also epistaticaly connected with two other regions: a genistein interaction with the locus Gm19/(*PAL*), and a glycitein interaction (Gm11) with a locus in the proximity of a QLT implicated in glycitein production [10,11]. A third strategic node in Gm07 accounts for genistein, daidzein and the sum of all isoflavones, and it is also connected with two other important areas in the genome. First, a daidzein epistatic interaction with the region in Gm01/(*CHS6*) of *qDAI1 *and a QTL reported for glycitein [11]. The second is an interaction for genistein with Gm13/(*IFS2*, *CHI3*), which itself also explains genistein seed concentrations in an additive manner. Three loci located in Gm02/(*F3H1*, *F3H2*)-Gm05-Gm15/(*I2'H*) formed another key three-node subnet for isoflavone synthesis. If a single genomic region harbors QTL for genistein, daidzein, glycitein and total isoflavones, it is certainly a good candidate to be considered for marker assisted selection. The underlying gene could be an enzyme acting early in the pathway or a *trans*-acting factor, which impacts expression or activity of one or more of these genes. One such region is found at the beginning of Gm02 corresponding to two *F3 H *genes (*F3H1 *and *F3H2*). This focal locus also featured two epistatic interactions accounting for daidzein and total isoflavones, respectively, with another locus in Gm05 for daidzein accumulation [10]. In addition, this Gm02 locus was also linked by means of an epistatic interaction for total isoflavones to a QTL in Gm15 for genistein, glycitein, and total seed isoflavone content, which is also connected with Gm10(*CHI*-*1B*). Finally, two other important additive nodes, in Gm04 (*qGEN4*, *qDAI4*, *qTOT4*) and Gm16 (*qDAI16*, *qTOT16*), were connected by a daidzein epistatic union.

Downstream and substrate-competing enzymes are less tractable to an intuitive interpretation, and likely require metabolite quantification to more precisely assess flux before considering their role as candidate genes. It is also difficult to establish a relationship with enzymes which govern reactions occurring far removed, or in another biosynthetic branch, from the QTL. This may be the case of for example the QTL region in Gm02 explaining the accumulation of all isoflavones (*qGEN2*, *qDAI2*, *qGLY2*, and *qTOT2*) and overlapping a copy of *F3H*. Other examples are: *IFR2 *for *qDAI4*, *qTOT4*, and *qGEN4 *in Gm04; *DFR2 *for *gly-D2_2 *in Gm17; *IOMT *for *gly-G *in Gm18; and *12'H *for *qGEN15*, *qGLY15*, and *qTOT15 *in Gm15. Product inhibition and other metabolic controls are likely involved and maintain the proportion of the different isoflavones within acceptable biochemical ranges.

Having placed the phenylpropanoid genes on the genetic map also offered us the possibility to formulate hypotheses on the genetic basis underlying epistasis. Surprisingly, a great number of the epistatic QTL have isoflavone synthesizing genes located in their IC (Figure 4), suggesting that a great number of the interacting loci could have a tractable genetic basis. Epistasis might be a critical factor for fitness-related traits in some plant species [62,63]. This is in perfect agreement with the role that isoflavones have in the fitness of soybean plants. However, epistatic QTL are subjected to dramatic adjustments, even more than their additive counterparts, when different environments are considered [12,14]. Despite these difficulties, researchers have successfully assigned genes to epistatic QTL that also had additive effects [44,64-66]. Finding the genes underlying epistatic QTL with no main-effect is more challenging but in some cases they have been revealed [67,68]. Trying to assess the complete network of isoflavone epistasis appears to be a colossal task because the number, environmentally-influenced, and small-effects of the interactions. Moreover, only genes on the phenylpropanoid pathway were placed on the linkage map, and although epistasis is more likely to occur between genes on the same or related pathways [69], undoubtedly interactions with other enzymes or transcription factors exist. Under these premises, one should be very cautious when intending to assign candidate genes to epistatic QTL. However, considering the genetic network as a whole might help not only in validating the individual components but also in deciphering genes underlying epistasis. For instance, if an epistatic locus also bears a main-effect, it may warrant further study. Moreover, if both epistatic and main-effect QTL account for the same trait it seems more likely not to be a spurious QTL. This extreme, frequently observed in our analysis, further validated many of the epistatic interactions, as opposed to being artifacts due to a limited sample size. For example, in Gm17 two independent main-effect QTL accounted for glycitein concentrations. Both had candidate genes in their IC, and both were found to interact with each other by an epistatic interaction, which accounted for glycitein accumulation itself.

## Conclusions

A large scale analysis was accomplished to identify genomic regions associated with isoflavone accumulation. We identified a number of such regions, which form a complex network controlling seed isoflavone levels Overall, our results suggest that isoflavone accumulation in soybean seeds is controlled by a complex environment-adaptable network of interacting nodes. The study clarifies, from a genetic point of view, why isoflavone concentration in soybean seeds is such a complex and variable trait. We could also place robust candidate genes for several main-effect loci in an attempt to find the gene lying beneath the QTL. Nevertheless, it remains for future research to determine the nature of the proposed allelic differences between candidate genes in the lines we examined, and the manner in which these differences correlate with impacts on isoflavone content of seeds. Validation of the entire network of interactions, however, is likely to remain a monumental task due to the numerous small-effect QTL involved and their environmental unpredictability. For breeding programs targeting seed isoflavones, the many QTL involved, their small percentage of the variance accounted for by each, at least in this population, and the complexity of the interactions do not forecast applying MAS with success.

## Methods

### Plant material and growing conditions

In a previous study we used a cross between Essex (low seed-isoflavone content) and the plant introduction PI 437654 (high seed-isoflavone content) to map isoflavone-content QTL [14]. The same 205 F_7_-derived recombinant inbred line (RIL) mapping population was planted in two-row plots in April of the following year (2007) at the same two locations: University of Missouri Bradford Research and Extension Center (BREC, 38°9'N) and the University of Missouri Delta Research Center (DRC, 36°44'N), with three replications per location. Plants were maintained under irrigated conditions until physiological maturity at which seed samples were harvested from a pool of at least three plants per RIL for each replication. Records of precipitation, temperatures, and other climatological parameters during the growing period for both locations and years can be found at http://aes.missouri.edu/bradford/weather/ and http://aes.missouri.edu/delta/weather/, respectively. Consequently, four different environments were used for the study, which consisted of 2 years (2006 and 2007), and 2 locations (BREC and DRC), and a total of 2460 (205×4×3) pooled seeds were phenotyped and later used as independent observations to perform an ample QTL mapping analysis.

### Isoflavone Extraction and Quantification

Genistein, daidzein, and glycitein amounts were determined as previously described [14]. Briefly, ~ 20 seeds from a pool of at least three plants per RIL for each replication were ground to a fine powder and extracted with 7 mL of 80% methanol at 55 °C for 2 h, vortexing every 30 min. The supernatant was cleaned using Fisherbrand 0.45 μm 25 mm nylon syringe filters (Fisher Scientific, Pittsburgh, PA). 10 μl of the filtered extraction was used for reverse-phase HPLC on an Agilent 1100 HPLC system (Santa Clara, CA). Separation and elution were performed by an 18 min linear gradient starting with 20% methanol/80% 10 mM ammonium acetate (v/v) (pH 5.6) and finishing with 100% methanol at 1 ml/min. A RP-C18 Lunar C2 column was used (Phenomenex, La Jolla, CA). Metabolites were detected by photodiode array. Identification and quantification of each isoflavone component were based on available standards (Indofine Chemical Co., Somerville, NJ).

### Statistical analysis, linkage map and QTL analysis

Statistical analysis was performed using the SAS STAT 9.1 program (SAS Institute Inc., Cary, NC). For the ANOVA, the pooled linear model contained the effect of environment, replication within environment, genotype, and environment × genotype. Effects were tested using PROC GLM. Heritability in the broad-sense over replicates and environments (H^2^) was calculated according to [70] for which the variance components were determined by the PROC GLM. The linkage map was previously described [71], and contained a total of 276 markers (SSR and AFLP) distributed on 26 linkage groups. The mixed-model based composite interval mapping implemented in QTLNetwork v2.0 (Institute of Bioinformatics, Zhejiang University, Hangzhou, China), and described in [72], was used for the QTL mapping analysis and run with two-year two-location input data and three replications per location. QTLNetwork, which was specifically developed for complex traits, can perform an integrated analysis using each replicate data as an independent entity, precluding the need to average, and thus allowing greater statistical power through larger data samples [41]. This mapping approach efficiently integrates effects of multiple QTLs, epistasis, and QTL-by-environment, by first conducting a whole genome scan for candidate marker intervals. The selected markers are subsequently used as cofactors for putative QTLs, followed by detection of significant marker-interval interactions. Finally, all is integrated by a whole-genome scan of epistasis conditioned on previously found QTLs and marker-interval interactions. Candidate interval selection, epistatic effects, and putative QTL detection were calculated with an experimental-wise Type I error of α = 0.05, α = 0.001, α = 0.001, respectively. QTL effects were estimated using Markov chain Monte Carlo method. Genome scan was performed using 10 cM window size and 1 cM walk speed. Critical F-value was assessed by permutation test using 1000 permutations rendering 7.4, 7.5, 7.2, and 8.0 for genistein, daidzein, glycitein, and total isoflavones, respectively. Individual replicated data and the four environments together were used to run QTLNetwork; however, data of each separate location and year were also used for comparison.

To calibrate the statistical power of the experimental design for detecting QTLs, a Monte Carlo simulation was conducted with the actual linkage map, sample size (2460 observations), and trait broad sense heritabilities, as previously reported [72]. For the simulations, a complex trait was supposed to be controlled by ten QTLs, with additive (A) and/or additive by environment interaction (A×E) effect components. One hundred simulations were run and the average estimates (± SE) were computed. Support intervals were calculated as described in [72]. Overall, simulations found a 100% power of detecting QTLs with relative contributions (RC) larger than 3.8%, a 68% with contributions larger than 1.5%, and a 58% larger than 0.8% (Additional file 10. Epistatic effects were not included in the simulations due to the overdemanding computational needs for their estimation with the aforementioned experimental parameters. However, including epistasis would likely further improve the statistical power to detect QTL as well as the false discovery rate estimates [14,73].

### Candidate gene identification

To discern the genomic location of candidate genes for isoflavone synthesis we assembled a list of NCBI gene entries derived from *Glycine max *and *Medicago truncatula*. The coding sequence of each entry was used as query in BLASTn searches, using an E-value cut off of 1.0E-05 against the Williams 82 Glyma1.01 sequence http://www.phytozome.net/. For one of these gene categories, chalcone synthase (*CHS*), we utilized tBLASTn with predicted protein sequences as query, with an E-value cut off of 1.0E-20. A list of gene models identified by BLAST searches were assembled and evaluated (Additional File 5). We assigned the best matching gene model the gene name corresponding to an NCBI entry (a list of all entries used is shown in Additional File 1), other matches are labeled "putative". Glyma1.01 gene model protein and nucleotide coding sequences were aligned to NCBI entries using AlignX software (Invitrogen, Carlsbad, CA) to determine protein and coding sequence % identity. Genetic map position for candidate genes were estimated by identifying the nearest flanking SSR or SNP genetic markers using the generic genome browser hosted on: http://www.soybase.org.

## Authors' contributions

JJGG conceived, designed and coordinated the study; also participated in isoflavone quantification, performed data analyses, prepared tables and figures, and wrote the manuscript. XW carried out genotyping analysis. JDG performed candidate gene analysis. JDL and JGS coordinated the field studies at Delta Research Center. RZ and OY participated in isoflavone quantification and data analyses. OY also provided valuable editorial advice. HTN contributed to editing of the manuscript and data analysis. ME accomplished the statistical analysis. DAS developed the mapping population, coordinated field studies, contributed to the conception and design of the study and editing of the manuscript. All authors read and approved the final manuscript.

## Supplementary Material

Additional file 1 **Individual trait distribution**. Distribution of values for genistein, daidzein, and glycitein measured at different environments. Click here for file

Additional file 2 **Additive by environment interaction effects**. Additive by environment interaction effect for genistein, daidzein, glycitein, and total isoflavone seed content. Click here for file

Additional file 3 **Additive by additive epistatic interactions**. Additive by additive epistatic interactions for genistein, daidzein, glycitein, and total isoflavones. Click here for file

Additional file 4 **Additive-additive by environment interaction effect for epistatic interactions**. Additive-additive by environment interaction effect for each epistatic interaction for genistein, daidzein, glycitein, and total isoflavones. Click here for file

Additional file 5 **List of gene models identified by BLAST searches**. Complete list of all genes included in this study with the flanking markers, gene models and BLAST parameters. Click here for file

Additional file 6 **Neighbor-joining phyllogenetic tree for chalcone synthase protein entries**. Neighbor-joining phyllogenetic tree generated using AlignX (Invitrogen) using NCBI soybean chalcone synthase protein entries. Click here for file

Additional file 7 **Neighbor-joining phyllogenetic tree for chalcone synthase coding sequence entries**. Neighbor-joining phyllogenetic tree generated using AlignX (Invitrogen) using NCBI soybean chalcone synthase coding sequence entries coding sequence. Click here for file

Additional file 8 **Protein alignment of chalcone synthase**. Protein alignment of NCBI soybean chalcone synthase VI protein sequence and the two putative CHS6 predicted predicted proteins present in Glyma1.01 Click here for file

Additional file 9 **Alignment of chalcone synthase coding region sequences**. Alignment of NCBI soybean chalcone synthase VI coding region sequence and the two putative CHS6 predicted coding regions present in Glyma1.01 Click here for file

Additional file 10 **Monte Carlo simulation results**. Summarized Monte Carlo simulation results for mapping QTLs with A and AxE effects. Click here for file

Additional file 11 **NCBI entries used for candidate gene identification**. Click here for file

## References

[B1] Cederroth CR Nef S Soy, phytoestrogens and metabolism: A review Mol Cell Endocrinol 2009 304 1-2 30 42 10.1016/j.mce.2009.02.027 19433245

[B2] Rochfort S Panozzo J Phytochemicals for health, the role of pulses J Agric Food Chem 2007 55 7981 7994 10.1021/jf071704w 17784726

[B3] Zhang J Yu O Hari Krishnan Metabolic engineering of isoflavone biosynthesis in seeds. *In *Modification of seed composition to promote health and nutrition 2009 Agronomy Monograph Series 151 177

[B4] Subramanian S Hu X Lu G Odelland JT Yu O The promoters of two isoflavone synthase genes respond differentially to nodulation and defense signals in transgenic soybean roots Plant Mol Biol 2004 54 623 639 10.1023/B:PLAN.0000040814.28507.35 15356384

[B5] Subramanian S Graham MY Yu O Graham TL RNA interference of soybean isoflavone synthase genes leads to silencing in tissues distal to the transformation site and to enhanced susceptibility to *Phytophthora sojae* Plant Physiol 2005 137 1345 1353 10.1104/pp.104.057257 15778457 PMC1088325

[B6] Subramanian S Stacey G Yu O Endogenous isoflavones are essential for the establishment of symbiosis between soybean and *Bradyrhizobium japonicum* Plant J 2006 48 261 273 10.1111/j.1365-313X.2006.02874.x 17018035

[B7] Subramanian S Stacey G Yu O Distinct, crucial roles of flavonoids during legume nodulation Trends Plant Sci 2007 12 282 285 10.1016/j.tplants.2007.06.006 17591456

[B8] Eldridge A Kwolek W Soybean isoflavones: Effect of the environment and variety on composition J Agric Food Chem 1983 31 394 396 10.1021/jf00116a052 6682871

[B9] Wang H Murphy PA Isoflavone Composition of American and Japanese Soybeans in Iowa: Effects of Variety, Crop Year, and Location J Agric Food Chem 1994 42 1674 1677 10.1021/jf00044a017

[B10] Kassem MA Meksem K Iqbal MJ Njiti VN Banz WJ Winters TA Wood A Lightfoot DA Definition of soybean genomic regions that control seed phytoestrogen amounts J Bio & Biotech 2004 1 52 60 10.1155/S1110724304304018 PMC545653 15123888

[B11] Kassem MA Shultz J Meksem K Cho Y Wood AJ Iqbal MJ Lightfoot DA An updated 'Essex' by 'Forrest' linkage map and first composite interval map of QTL underlying six soybean traits Theor Appl Genet 2006 113 1015 1026 10.1007/s00122-006-0361-8 16953420

[B12] Primomo VS Poysa V Ablett GR Jackson CJ Gijzen M Rajcan I Mapping QTL for individual and total isoflavone content in soybean seeds Crop Sci 2005 45 2454 2462 10.2135/cropsci2004.0672

[B13] Zeng G Li D Han Y Teng W Wang J Qiu L Li W Identification of QTL underlying isoflavone contents in soybean seeds among multiple environments Theor Appl Genet 2009 118 1455 1463 10.1007/s00122-009-0994-5 19266178

[B14] Gutierrez-Gonzalez JJ Wu X Zhang J Lee JD Ellersieck M Shannon JG Yu O Nguyen HT Sleper DA Genetic control of soybean seed isoflavone content: Importance of statistical model and epistasis in complex traits Theor Appl Genet 2009 119 6 1069 1083 10.1007/s00122-009-1109-z 19626310 PMC2755750

[B15] Schmutz J Cannon SB Schlueter J Ma J Mitros T Nelson W Hyten DL Song Q Thelen JJ Cheng J Xu D Hellsten U May GD Yu Y Sakurai T Umezawa T Bhattacharyya MK Sandhu D Valliyodan B Lindquist E Peto M Grant D Shu S Goodstein D Barry K Futrell-Griggs M Abernathy B Du J Tian Z Zhu L Gill N Joshi T Libault M Sethuraman A Zhang XC Shinozaki K Nguyen HT Wing RA Cregan P Specht J Grimwood J Rokhsar D Stacey G Shoemaker RC Jackson SA Genome sequence of the palaeopolyploid soybean Nature 2010 463 178 183 10.1038/nature08670 20075913

[B16] Tsukamoto C Shimada S Igita K Kudou S Kokubun M Okubo K Kitamura K Factors affecting isoflavone content in soybean seeds: Changes in isoflavones, saponins, and composition of fatty acids at different temperatures during seed development J Agric Food Chem 1995 43 1184 1192 10.1021/jf00053a012

[B17] Stafford HA Roles of flavonoids in symbiotic and defense functions in legume roots Bot Rev 1997 63 27 39 10.1007/BF02857916

[B18] Dhaubhadel S McGarvey BD Williams R Gijzen M Isoflavonoid biosynthesis and accumulation in developing soybean seeds Plant Mol Biol 2003 53 733 743 10.1023/B:PLAN.0000023666.30358.ae 15082922

[B19] Bennett JO Yu O Heatherly LG Krishnan HB Accumulation of genistein and daidzein, soybean isoflavones implicated in promoting human health, is significantly elevated by irrigation J Agric Food Chem 2004 52 7574 7579 10.1021/jf049133k 15675806

[B20] Lozovaya VV Lygin AV Ulanov AV Nelson RL Dayde J Widhohm JM Effect of temperature and soil moisture status during seed development on soybean seed isoflavone concentration and composition Crop Sci 2005 45 1934 1940 10.2135/cropsci2004.0567

[B21] Hoeck JA Fehr WR Murphy PA Welke GA Influence of genotype and environment on isoflavone contents of soybean Crop Sci 2000 40 48 51 10.2135/cropsci2000.40148x

[B22] Mebrahtu T Mohamed A Wang CY Andebrhan T Analysis of isoflavone contents in vegetable soybeans Plant Foods for Human Nutrition 2004 59 55 61 10.1007/s11130-004-0023-4 15678752

[B23] Murphy SE Lee EA Woodrow L Seguin P Kumar J Rajcan I Ablett GR Genotype × Environment interaction and stability for isoflavone content in soybean Crop Sci 2009 49 1313 1321 10.2135/cropsci2008.09.0533

[B24] Yu O McGonigle B Metabolic engineering of isoflavone biosynthesis Advances in Agronomy 2005 86 147 190 full_text

[B25] Carlborg O Haley CS Epistasis: too often neglected in complex trait studies? Nature reviews 2004 5 618 625 10.1038/nrg1407 15266344

[B26] Doerge RW Mapping and analysis of quantitative trait loci in experimental populations Nature Reviews 2001 3 43 52 10.1038/nrg703 11823790

[B27] Phillips PC Epistasis, the essential role of gene interactions in the structure and evolution of genetic systems Nature Reviews 2008 9 855 867 10.1038/nrg2452 18852697 PMC2689140

[B28] Wright S Evolution in Mendelian populations Genetics 1931 16 97 159 17246615 10.1093/genetics/16.2.97 PMC1201091

[B29] Doebley J Stec A Gustus C Teosinte branched1 and the origin of maize: evidence for epistasis and the evolution of dominance Genetics 1995 141 333 346 8536981 10.1093/genetics/141.1.333 PMC1206731

[B30] Whitlock MC Multiple fitness peaks and epistasis Annu Rev Ecol Syst 1995 26 601 629 10.1146/annurev.es.26.110195.003125

[B31] Weinreich DM Watson RA Chao L Perspective: sign epistasis and genetic constraint on evolutionary trajectories Evolution 2005 59 1165 1174 16050094

[B32] Malmberg RL Mauricio R QTL-based evidence for the role of epistasis in evolution Genet Res Camb 2005 86 89 95 10.1017/S0016672305007780 16356282

[B33] Melchinger AE Piepho HP Utz HF Muminovic J Wegenast TW Torjek O Altmann T Kusterer B Genetic basis of heterosis for growth-related traits in Arabidopsis investigated by testcross progenies of near-isogenic lines reveals a significant role of epistasis Genetics 2007 177 1827 1837 10.1534/genetics.107.080564 18039884 PMC2147963

[B34] Sanjuan R Elena SF Epistasis correlates to genomic complexity PNAS 2006 103 14402 14405 10.1073/pnas.0604543103 16983079 PMC1599975

[B35] Wilfert L Schmid-Hempel P The genetic architecture of susceptibility to parasites BMC Evolutionary Biology 2008 8 187 1994 10.1186/1471-2148-8-187 18590517 PMC2446395

[B36] Shoemaker RC Sculueter JA Jackson SA Soybean Genome Structure and Organization Book chapter in Genetics and Genomics of Soybean G. Stacey (ed.) 2008 Springer Science+Business Media LLC

[B37] Shoemaker RC Schlueter J Doyle JJ Paleopolyploidy and gene duplication in soybean and other legumes Current Opinion in Plant Biology 2006 9 104 109 10.1016/j.pbi.2006.01.007 16458041

[B38] Doyle JJ Flagel LE Paterson HA Rapp RA Soltis DE Soltis PS Wendel JF Evolutionary Genetics of Genome Merger and Doubling in Plants Annual Review of Genetics 2008 42 443 461 10.1146/annurev.genet.42.110807.091524 18983261

[B39] Koes RE Quattrocchio F King AA The flavonoid biosynthetic pathway in plants-Function and evolution Bioessays 1994 16 123 132 10.1002/bies.950160209

[B40] Zernova OV Lygin AV Widholm JM Lozovaya VV Modification of isoflavones in soybean seeds via expression of multiple phenolic biosynthetic genes Plant Physiol Biochem 2009 47 769 777 10.1016/j.plaphy.2009.05.006 19539487

[B41] Wang DL Zhu J Paterson AH Mapping QTLs with epistatic effects and QTL×environment interactions by mixed linear model approaches Theor Appl Genet 1999 99 1255 1264 10.1007/s001220051331

[B42] Bernardo R Molecular markers and selection for complex traits in plants: learning from the last 20 years Crop Sci 2008 48 1649 1664 10.2135/cropsci2008.03.0131

[B43] Melchinger AE Utz HF Schon CC Quantitative trait locus (QTL) mapping using different testers and independent population samples in maize reveals low power of QTL detection and large bias in estimates of QTL effects Genetics 1998 149 383 403 9584111 10.1093/genetics/149.1.383 PMC1460144

[B44] Alcazar R Garcia AV Parker JE Reymond M Incremental steps toward incompatibility revealed by Arabidopsis epistatic interactions modulating salicylic acid pathway activation PNAS 2009 106 334 339 10.1073/pnas.0811734106 19106299 PMC2629243

[B45] Beggs CJ Stolzer-Jehle A Wellmann E Isoflavonoid formation as an indicator of UV stress in bean (*Phaseolus vulgaris *L.) leaves: the significance of photorepair in assessing potential damage by increased solar UV-B radiation Plant Physiol 1985 79 3 630 634 10.1104/pp.79.3.630 16664463 PMC1074942

[B46] Variyar PS Limaye A Sharma A Radiation-induced enhancement of antioxidant contents of soybean (*Glycine max *Merrill) J Agric Food Chem 2004 52 3385 3388 10.1021/jf030793j 15161202

[B47] Naoumkina M Farag MA Sumner LW Tang Y Liu CJ Dixon RA Different mechanisms for phytoalexin induction by pathogen and wound signals in *Medicago truncatula* PNAS 2007 104 17909 17915 10.1073/pnas.0708697104 17971436 PMC2084270

[B48] Beavis WD The power and deceit of QTL experiments: lessons from comparative QTL studiesproceedings of the 49th annual corn and sorghum industry research conference Washington DC 1994

[B49] Xu S Theoretical basis of the Beavis effect Genetics 2003 165 2259 2268 14704201 10.1093/genetics/165.4.2259 PMC1462909

[B50] Kearsey MJ Pooni HS Syed NH Genetics of quantitative traits in Arabidopsis thaliana Heredity 2003 91 45 464 10.1038/sj.hdy.6800306 14576738

[B51] Goodnight CJ Epistasis and the effect of founder events on the additive genetic variance Evolution 1988 42 441 454 10.2307/2409030 28564006

[B52] Ahrent DK Caviness CE Natural cross-pollination of twelve soybean cultivars in Arkansas Crop Sci 1994 34 376 378 10.2135/cropsci1994.0011183X003400020013x

[B53] Holland JB Genetic architecture of complex traits in plants Current Opinion in Plant Biology 2007 10 156 161 10.1016/j.pbi.2007.01.003 17291822

[B54] Rowe HC Hansen BG Halkier BA Kliebenstein DJ Biochemical networks and epistasis shape the Arabidopsis thaliana metabolome Plant Cell 2008 20 1199 1216 10.1105/tpc.108.058131 18515501 PMC2438456

[B55] Otto SP Jones CD Detecting the undetected: Estimating the total number of loci underlying a quantitative trait Genetics 2000 156 2093 2107 11102398 10.1093/genetics/156.4.2093 PMC1461347

[B56] Gutierrez-Gonzalez JJ Guttikonda S Aldrich DL Tran LSP Zhong R Yu O Nguyen HT Sleper DA Differential expression of isoflavone biosynthetic genes in soybean during water deficits Plant & Cell Physiol 2010 in press 20430761 10.1093/pcp/pcq065

[B57] Matsumura H Watanabe S Harada K Senda M Akada S Kawasaki S Dubouzet EG Minaka N Takahashi R Molecular linkage mapping and phylogeny of the chalcone synthase multigene family in soybean Theor Appl Genet 2005 110 1203 1209 10.1007/s00122-005-1950-7 15791451

[B58] Tuteja JH Vodkin LO Structural features of the endogenous CHS silencing and target loci in the soybean genome The Plant Genome Crop Sci 2008 48 S1 S49 S68

[B59] Todd JJ Vodkin LO Duplications that suppress and deletions that restore expression from a chalcone synthase multigene family Plant Cell 1996 8 687 699 10.1105/tpc.8.4.687 12239396 PMC161129

[B60] Kasai A Watarai M Yumoto S Akada S Ishikawa R Harada T Niizeki M Senda M Influence of PTGS on Chalcone Synthase Gene Family in Yellow Soybean Seed Coat Breed Sci 2004 54 355 360 10.1270/jsbbs.54.355

[B61] Tuteja JH Clough SJ Chan WC Vodkin LO Tissue-specific gene silencing mediated by a natural occurring chalcone synthase gene cluster in *Glycine max* Plant Cell 2004 16 819 835 10.1105/tpc.021352 15064367 PMC412859

[B62] Mei HW Li ZK Shu QY Guo LB Wang YP Yu XQ Ying CS Luo LJ Gene actions of QTLs affecting several agronomic traits resolved in a recombinant inbred rice population and two backcross populations Theor Appl Genet 2003 107 89 101 12721635 10.1007/s00122-003-1192-5

[B63] Malmberg RL Held S Waits A Mauricio R Epistasis for fitness-related quantitative traits in Arabidopsis thaliana grown in the field and in the greenhouse Genetics 2005 171 2013 2027 10.1534/genetics.105.046078 16157670 PMC1456117

[B64] McMullen MD Snook M Lee EA Byrne PF Kross H Musket TA Houchins K Coe EH Jr The biological basis of epistasis between quantitative trait loci for flavones and 3-deoxyanthocyanin synthesis in maize (*Zea mays *L.) Genome 2001 44 667 676 10.1139/gen-44-4-667 11550903

[B65] Kroymann J Mitchell-Olds T Epistasis and balanced polymorphism influencing complex trait variation Nature 2005 435 95 98 10.1038/nature03480 15875023

[B66] Sweigart AL Fishman L Willis JH A simple genetic incompatibility causes hybrid male sterility in mimulus Genetics 2006 172 2465 2479 10.1534/genetics.105.053686 16415357 PMC1456371

[B67] Lark KG Chase K Adler F Mansur LM Orf JH Interactions between quantitative trait loci in soybean in which trait variation at one locus is conditional upon a specific allele at another PNAS 1995 92 4656 4660 10.1073/pnas.92.10.4656 7753859 PMC42003

[B68] Ehrenreich IM Stafford PA Purugganan MD The genetic architecture of shoot branching in Arabidopsis thaliana: a comparative assessment of candidate gene associations vs. quantitative trait locus mapping Genetics 2007 176 1223 1236 10.1534/genetics.107.071928 17435248 PMC1894586

[B69] Segre D DeLuna A Church GM Kishony R Modular epistasis in yeast metabolism Nat Genet 2005 37 77 83 15592468 10.1038/ng1489

[B70] Hill J Becker HC Tigerstedt PMA Quantitative and ecological aspects of plant breeding 1998 Chapman and Hall, London

[B71] Wu X Blake S Sleper DA Shannon G Cregan P Nguyen HT QTL, additive and epistatic effects for SCN resistance in PI 437654 Theor Appl Genet 2008 118 1093 1105 10.1007/s00122-009-0965-x 19184662

[B72] Yang J Zhu J Williams RW Mapping the genetic architecture of complex traits in experimental populations Bioinformatics 2007 23 1527 1536 10.1093/bioinformatics/btm143 17459962

[B73] Yi N Xu S Mapping quantitative trait loci with epistatic effects Genet Res Camp 2002 79 185 198 10.1017/s0016672301005511 12073556

