# Supplementary material for: Intricate environment-modulated genetic networks control isoflavone accumulation in soybean seeds
Source: BMC Plant Biol. 2010 Jun 11;10:105. doi: 10.1186/1471-2229-10-105 (PMC3224685; doi:10.1186/1471-2229-10-105)
Supplement: Additional file 1 — Individual trait distribution. Distribution of values for genistein, daidzein, and glycitein measured at different environments. [file 1471-2229-10-105-S1.DOC]

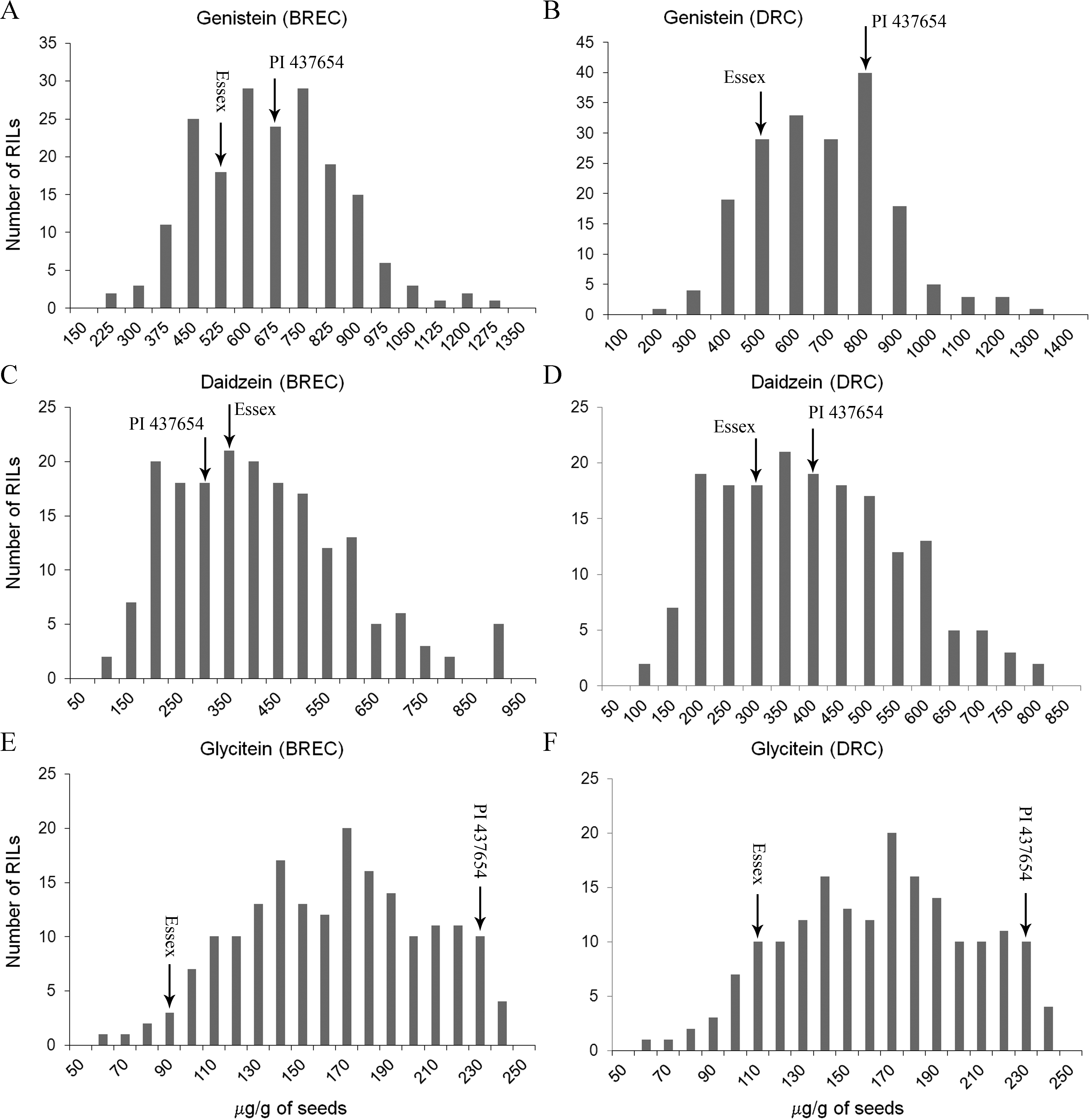


**Additional File 1**. Distribution of average genistein (A, B), daidzein (C, D), and glycitein (E, F), in three replications of Essex × PI 437654 RILs growing in two field locations, BREC (A, C, E) and DRC (B, D, F) in year 2007 (for year 2006 data see Gutierrez-Gonzalez et al 2009 – reference [14]). Arrows indicate the position of the two parental lines. Horizontal axis shows each particular isoflavone seed content in μg/g of seeds.
