# Supplementary material for: Intricate environment-modulated genetic networks control isoflavone accumulation in soybean seeds
Source: BMC Plant Biol. 2010 Jun 11;10:105. doi: 10.1186/1471-2229-10-105 (PMC3224685; doi:10.1186/1471-2229-10-105)
Supplement: Additional file 2 — Additive by environment interaction effects. Additive by environment interaction effect for genistein, daidzein, glycitein, and total isoflavone seed content. [file 1471-2229-10-105-S2.DOC]

| Additional File 2. Additive by environment interaction effect for genistein, daidzein, glycitein, and total isoflavone seed content. | | | | | | | | | | |
| --- | --- | --- | --- | --- | --- | --- | --- | --- | --- | --- |
| Name^a^ | Interval^b^ | AE1±SE | P-Value | AE2±SE | P-Value | AE3±SE | P-Value | AE4±SE | P-Value | h^2^(ae) |
| Genistein |  |  |  |  |  |  |  |  |  |  |
| *qGEN5* | SATT236-SAT_271 | **27.9±10** | **0.005461** | **-24.6±9.9** | **0.013538** | **28.2±9.9** | **0.004260** | **-31.9±9.8** | **0.001175** | **0.7** |
| *qGEN2* | SAT_279-EACAMCTT123 | 3.0±8.0 | 0.708024 | 6.7±7.9 | 0.394524 | **15.6±7.9** | **0.046630** | **-25.2±7.8** | **0.001260** | **0.3** |
| *qGEN15* | SAT_112-SATT691 | -0.0±0.1 | 0.999235 | 0.0±0.1 | 0.996445 | 0.0±0.1 | 0.999592 | 0.0±0.1 | 0.996845 | 0.0 |
| *qGEN13* | SATT490-SAT_197 | -0.6±4.3 | 0.882951 | 1.8±4.3 | 0.685061 | 2.1±4.3 | 0.615019 | -3.3±4.3 | 0.444743 | 0.2 |
| *qGEN12* | SCTT009-SATT541 | 13.6±7.3 | 0.064538 | -13.5±7.3 | 0.063109 | 1.3±7.2 | 0.858243 | -1.0±7.2 | 0.886557 | 0.2 |
| *qGEN20* | GMLPSI2-SCT_189 | -4.2±5.1 | 0.403778 | -0.1±5.0 | 0.991082 | 4.8±5.0 | 0.338265 | -0.6±5.0 | 0.906994 | 0.0 |
| *qGEN19* | SAT_113-SAT_286 | **33.6±8.6** | **0.000101** | **18.2±8.5** | **0.032716** | 8.8±8.5 | 0.295733 | 6.4±8.4 | 0.448362 | **0.3** |
| *qGEN7* | SATT175-EAGGMCTT095 | 0.0±0.0 | 0.996859 | 0.0±0.1 | 0/997755 | 0.0±0.1 | 0.998125 | 0.0±0.1 | 0.997130 | 0.0 |
| Daidzein |  |  |  |  |  |  |  |  |  |  |
| *qDAI5* | SATT174-SATT236 | 0.0±0.1 | 0.998503 | 0.0±0.1 | 0.990296 | 0.0±0.1 | 0.994541 | 0.0±0.1 | 0.997356 | 0.1 |
| *qDAI8* | SATT187-EAGGMCTT205 | 0.0±0.1 | 0.992809 | 0.0±0.1 | 0.993700 | 0.0±0.1 | 0.998835 | 0.0±0.1 | 0.998109 | 0.0 |
| *qDAI4* | SATT396-SAT_337 | 0.5±5.1 | 0.919324 | 8.3±5.0 | 0.101241 | -1.3±5.0 | 0.790569 | -7.5±5.0 | 0.137302 | 0.1 |
| *qDAI1* | SAT_106-AW781285 | 0.0±0.1 | 0.993312 | 0.0±0.1 | 0.993444 | 0.0±0.1 | 0.993930 | 0.0±0.1 | 0.993771 | 0.0 |
| *qDAI2* | SAT_279-EACAMCTT123 | 1.5±5.8 | 0.800912 | 5.9±5.8 | 0.306583 | 10.1±5.7 | 0.079629 | **-17.6±5.7** | **0.002088** | **0.3** |
| *qDAI12* | SCTT009-SATT541 | 9.7±5.6 | 0.085316 | **-12.6±5.5** | **0.022917** | 2.2±5.5 | 0.684157 | 0.4±5.5 | 0.939194 | **0.2** |
| *qDAI16* | SAT_339-SATT280 | -2.6±7.1 | 0.715517 | 5.6±7.0 | 0.430061 | **-17.4±7.0** | **0.013073** | **14.6±7.0** | **0.036985** | **0.3** |
| *qDAI7* | SATT175-EAGGMCTT095 | 2.9±5.6 | 0.599713 | -3.6±5.6 | 0.521206 | -5.6±5.6 | 0.312678 | 6.3±5.5 | 0.254512 | 0.1 |
| *qDAI7_2* | SAT_147-SAT_330 | 3.4±5.1 | 0.508167 | 3.7±5.0 | 0.465528 | **-10.6±5.0** | **0.034565** | 3.4±5.0 | 0.495523 | **0.2** |
| Glycitein |  |  |  |  |  |  |  |  |  |  |
| *qGLY5* | SATT236-SAT_271 | 0.1±2.2 | 0.966555 | **-8.6±2.1** | **0.000067** | **11.6±2.1** | **0.000000** | -2.7±2.1 | 0.208749 | **1.1** |
| *qGLY6* | SATT281-SATT291 | **-4.3±1.6** | **0.008795** | 0.2±1.6 | 0.886513 | 1.5±1.6 | 0.331807 | 2.5±1.6 | 0.108098 | **0.4** |
| *qGLY6_2* | SATT319-EAACMCAC113 | 1.0±1.8 | 0.591473 | **-4.8±1.8** | **0.006132** | -0.4±1.7 | 0.836391 | **4.3±1.7** | **0.014656** | **0.3** |
| *qGLY2* | SAT_279-EACAMCTT123 | **-6.8±1.7** | **0.000118** | -1.3±1.7 | 0.433600 | **8.8±1.7** | **0.000000** | -0.8±1.7 | 0.639984 | **0.7** |
| *qGLY15* | SAT_112-SATT691 | 1.7±1.6 | 0.297408 | **-3.2±1.6** | **0.044519** | 0.4±1.6 | 0.803174 | 1.1±1.6 | 0.482644 | **0.1** |
| *qGLY9* | SATT242-EAACMCAC227 | 0.0±0.0 | 0.999675 | 0.0±0.0 | 0.987415 | 0.0±0.0 | 0.995668 | 0.0±0.0 | 0.991592 | 0.0 |
| Total |  |  |  |  |  |  |  |  |  |  |
| *qTOT5* | SATT174-SATT236 | 16.9±15.3 | 0.267543 | **-34.5±15.2** | **0.022955** | **39.2±15.1** | **0.009531** | -20.6±15.0 | 0.170330 | **0.4** |
| *qTOT4* | SAT_337-SAT_140 | -2.0±8.9 | 0.821352 | 7.0±8.9 | 0.429094 | 0.5±8.8 | 0.959430 | -4.6±8.8 | 0.600221 | 0.0 |
| *qTOT2* | SAT_279-EACAMCTT123 | -2.0±15.2 | 0.895228 | 13.2±15.0 | 0.381580 | **35.6±15.0** | **0.017396** | **-46.2±14.9** | **0.001968** | **0.4** |
| *qTOT2_2* | SAT_139-SAT_289 | -2.0±11.4 | 0.860260 | 17.0±11.3 | 0.131531 | -9.5±11.3 | 0.401198 | -5.6±11.2 | 0.617930 | 0.2 |
| *qTOT15* | SAT_112-SATT691 | 0.0±0.1 | 0.999522 | 0.0±0.1 | 0.999205 | 0.0±0.1 | 0.999694 | 0.0±0.1 | 0.999027 | 0.0 |
| *qTOT12* | SCTT009-SATT541 | 17.8±13.8 | 0.197416 | **-30.0±13.6** | **0.027618** | 15.8±13.6 | 0.245374 | -3.9±13.5 | 0.770567 | **0.3** |
| *qTOT16* | SAT_339-SATT280 | -11.5±19.8 | 0.559741 | 11.2±19.5 | 0.565313 | **-40.1±19.5** | **0.039933** | **40.1±19.4** | **0.038810** | **0.3** |
| *qTOT19* | SAT_286-SATT229 | **-29.4±14.8** | **0.047158** | 19.2±14.6 | 0.190867 | 14.9±14.6 | 0.305733 | -4.1±14.6 | 0.777545 | **0.2** |
| *qTOT7* | SATT175-EAGGMCTT095 | 0.0±0.1 | 0.999572 | 0.0±0.1 | 0.998205 | 0.0±0.1 | 0.997663 | 0.0±0.1 | 0.999853 | 0.0 |
| ^a^Name given to a particular QTL, *gen, dai, gly,* and *tot,* for genistein, daidzein, glycitein, and total isoflavone seed content, respectively, followed by the chromosome number and a number when more than one in the same chromosome. ^b^Interval of confidence in centiMorgans with respect to the first marker in the LG. AE1, AE2, AE3, and AE4 are the environment × interaction effect in μg/g plus/minus standard error for BREC_06 (AE1), DRC_06 (AE2),BREC_07 (AE3), and DRC_07 (AE4). h^2^(ae) is the heritability of the additive by environment interaction effects. P-values represent the significance of each effect. | | | | | | | | | | |
