# Supplementary material for: Intricate environment-modulated genetic networks control isoflavone accumulation in soybean seeds
Source: BMC Plant Biol. 2010 Jun 11;10:105. doi: 10.1186/1471-2229-10-105 (PMC3224685; doi:10.1186/1471-2229-10-105)
Supplement: Additional file 3 — Additive by additive epistatic interactions. Additive by additive epistatic interactions for genistein, daidzein, glycitein, and total isoflavones. [file 1471-2229-10-105-S3.DOC]

| Additional File 3. Additive by additive epistatic interactions for genistein, daidzein, glycitein, and total isoflavones. | | | | |
| --- | --- | --- | --- | --- |
| interval_i^a^ | interval_j^a^ | AA±SE^b^ | P-Value | h^2(aa)^c^ |
| *Genistein* |  |  |  |  |
| SATT236-SAT_271 | SATT187-EAGGMCTT205 | 24.9±5.2 | 0.000002 | 0.6 |
| SATT187-EAGGMCTT205 | SAT_113-SAT_286 | -15.3±4.5 | 0.000626 | 0.8 |
| SATT490-SAT_197 | SATT175-EAGGMCTT095 | -31.5±5.2 | 0.000000 | 1.8 |
| EAAGMCAT279-SATT050 | SATT484-AQ851479 | 23.3±4.5 | 0.000000 | 0.5 |
| SATT050-SATT385 | SATT643-SATT319 | 48.7±6.9 | 0.000000 | 0.4 |
| SATT187-EAGGMCTT205 | SATT270-SAT_268 | -23±4.3 | 0.000000 | 0.6 |
| SATT233-SATT329 | SATT484-AQ851479 | 32.2±4.6 | 0.000000 | 0.8 |
| AW620774-SATT534 | EAGGMCTT143-SATT182 | -24.1±5.7 | 0.000024 | 0.4 |
| EAACMCTC178-SATT578 | SAT_222-EACAMCTT122 | 36.1±4.6 | 0.000000 | 1.4 |
| SATT184-SAT_353 | GMLPSI2-SCT_189 | -27.7±4.1 | 0.000000 | 0.9 |
| EACAMCTT123-BE475343 | SATT346-SAT_147 | -14.8±4.8 | 0.002152 | 0.3 |
| SATT634-SATT266 | EAACMCAC175-SATT244 | 23.8±4.8 | 0.000001 | 1.1 |
| SATT634-SATT266 | EAACMCTC276-SCTT011 | 20.9±6.1 | 0.000661 | 0.1 |
| SCT_189-SATT440 | SATT435-SATT323 | -33.6±4.2 | 0.000000 | 1.1 |
| *Daidzein* |  |  |  |  |
| SATT174-SATT236 | SATT187-EAGGMCTT205 | 20.8±3.2 | 0.000000 | 0.6 |
| SATT187-EAGGMCTT205 | SATT175-EAGGMCTT095 | 16.7±3.5 | 0.000002 | 0.9 |
| SATT396-SAT_337 | SAT_339-SATT280 | 15.9±4.2 | 0.000201 | 0.6 |
| SAT_106-AW781285 | SATT175-EAGGMCTT095 | -18.7±3.7 | 0.000001 | 1.1 |
| SATT591-AZ536570 | SAT_279-EACAMCTT123 | 14.9±3.2 | 0.000003 | 0.7 |
| SAT_272-SAT_411 | SATT674-SAT_228 | -23.3±3.1 | 0.000000 | 1.2 |
| SATT359-EACAMCTT067 | SATT309-RHG1_INDEL | 18.4±3.5 | 0.000000 | 1.1 |
| SAT_342-EAACMCTC384 | SAT_380-SATT263 | 17.2±3.5 | 0.000001 | 1.0 |
| SAT_062-SATT281 | EAACMCAC175-SATT244 | 23±3.3 | 0.000000 | 0.7 |
| EAACMCTC063-EACAMCTT419 | SATT302-SATT142 | 49.5±3.8 | 0.000000 | 3.6 |
| EAACMCTC063-EACAMCTT419 | SATT522-AW756935 | 14.4±3.4 | 0.000033 | 0.3 |
| SATT266-SAT_135 | SAT_282-SAT_242 | 38.9±4 | 0.000000 | 0.9 |
| SAT_333-SCT_192 | SAT_284-SATT372 | 39.9±3.2 | 0.000000 | 1.3 |
| EACAMCTT122-SATT082 | SATT167-SATT552 | 39.1±3.3 | 0.000000 | 2.8 |
| SATT691-SATT598 | SAT_190-SCAA001 | -23.1±4.3 | 0.000000 | 0.6 |
| SAT_380-SATT263 | EAGGMCTT098-SATT009 | 29±3.3 | 0.000000 | 1.9 |
| SAT_380-SATT263 | SATT490-SAT_197 | 28±3.2 | 0.000000 | 0.4 |
| EAGGMCAG365-EAGGMCAG422 | SATT522-AW756935 | -26.1±3 | 0.000000 | 1.4 |
| SATT242-EAACMCAC227 | SATT339-SATT257 | -11.6±3.3 | 0.000585 | 0.2 |
| EAGGMCTT176-EAGGMCT135 | SATT683-SAT_275 | 21.4±4.2 | 0.000000 | 0.4 |
| SAT_121-SATT346 | SATT259-SATT188 | -31.9±4 | 0.000000 | 1.0 |
| *Glycitein* |  |  |  |  |
| SATT385-SATT619 | SAT_389-SATT636 | 7.2±0.9 | 0.000000 | 1.2 |
| EAACMCAC086-SATT187 | SAT_247-SATT519 | -4.4±1.1 | 0.000028 | 0.3 |
| SATT304-SATT416 | EACAMCTT122-SATT082 | 2.4±1 | 0.014723 | 0.3 |
| EAACMCTC178-SATT578 | EAACMCTC379-SATT316 | 4.6±0.9 | 0.000001 | 0.6 |
| SAT_135-SATT546 | SAT_284-SATT372 | 8.6±1.1 | 0.000000 | 1.2 |
| SATT458-SAT_284 | SAT_086-SATT256 | 6.5±1 | 0.000000 | 1.0 |
| *Total isoflavones* |  |  |  |  |
| SAT_279-EACAMCTT123 | SAT_112-SATT691 | -60.8±9.1 | 0.000000 | 1.6 |
| SAT_265-EAAGMCAT279 | SAT_279-EACAMCTT123 | 54.3±9.9 | 0.000000 | 1.0 |
| SATT089-SATT233 | SAT_275-SATT387 | 109.8±10.2 | 0.000000 | 2.1 |
| SATT455-SATT228 | SATT304-SATT416 | 77.2±9.1 | 0.000000 | 1.2 |
| SATT281-SATT291 | AW756935-SAT_090 | 62.5±7.9 | 0.000000 | 2.1 |
| SCT_033-SCT_188 | SATT683-SAT_275 | 53.2±8.7 | 0.000000 | 0.6 |
| SATT292-SAT_419 | EAACMCTC276-SCTT011 | 66.4±11.2 | 0.000000 | 1.1 |
| SCT_189-SATT440 | SCT_195-SATT159 | 80.1±7.8 | 0.000000 | 2.4 |
| ^a^Marker interval within each epistatic interaction occurs. ^b^Estimated additive by additive effect ± standard error, a positive sign for epistatic effects indicate that parental allele combinations at the two loci involved in epistasis increase each particular isoflavones expression while a negative sign indicate that recombinant allele combinations increase total isoflavone values. ^c^Heritability of the additive by additive interaction effect (%). P-values represent the significance of each effect. | | | | |
