# Supplementary material for: Intricate environment-modulated genetic networks control isoflavone accumulation in soybean seeds
Source: BMC Plant Biol. 2010 Jun 11;10:105. doi: 10.1186/1471-2229-10-105 (PMC3224685; doi:10.1186/1471-2229-10-105)
Supplement: Additional file 4 — Additive-additive by environment interaction effect for epistatic interactions. Additive-additive by environment interaction effect for each epistatic interaction for genistein, daidzein, glycitein, and total isoflavones. [file 1471-2229-10-105-S4.DOC]

| Additional File 4. Additive-additive by environment interaction effect for each epistatic interaction for genistein, daidzein, glycitein, and total isoflavones. | | | | | | | | | | |
| --- | --- | --- | --- | --- | --- | --- | --- | --- | --- | --- |
| interval_i^a^ | interval_j^a^ | AAE1±SE^b^ | P-Value | AAE2±SE^b^ | P-Value | AAE3±SE^b^ | P-Value | AAE4±SE^b^ | P-Value | h^2^(aae)^c^ |
| *Genistein* |  |  |  |  |  |  |  |  |  |  |
| SATT236-SAT_271 | SATT187-EAGGMCTT205 | 4.9±6.4 | 0.438456 | 0.9±6.3 | 0.892393 | -6.0±6.3 | 0.338042 | 0.2±6.3 | 0.968967 | 0.0 |
| SATT187-EAGGMCTT205 | SAT_113-SAT_286 | **-18.2±8** | **0.022991** | -1.6±7.9 | 0.838668 | 12.2±7.9 | 0.121957 | 7.4±7.9 | 0.348758 | **0.3** |
| SATT490-SAT_197 | SATT175-EAGGMCTT095 | 2.2±4.3 | 0.618113 | 0.5±4.3 | 0.909970 | -2.4±4.3 | 0.576238 | -0.3±4.3 | 0.951401 | 0.3 |
| EAAGMCAT279-SATT050 | SATT484-AQ851479 | 0.2±8.4 | 0.979026 | -13.9±8.3 | 0.094378 | **21.3±8.2** | **0.009923** | -7.8±8.2 | 0.345357 | **0.1** |
| SATT050-SATT385 | SATT643-SATT319 | -10.0±11.5 | 0.371531 | 12.3±11.4 | 0.282247 | 5.1±11.4 | 0.652100 | -7.4±11.3 | 0.516764 | 0.0 |
| SATT187-EAGGMCTT205 | SATT270-SAT_268 | 2.9±5.6 | 0.602239 | -3.7±5.6 | 0.508114 | -3.0±5.5 | 0.583197 | 3.8±5.4 | 0.489654 | 0.0 |
| SATT233-SATT329 | SATT484-AQ851479 | 0.0±0.1 | 0.998606 | 0.0±0.1 | 0.998307 | 0.0±0.1 | 0.998869 | 0.0±0.1 | 0.998134 | 0.0 |
| AW620774-SATT534 | EAGGMCTT143-SATT182 | 2.1±10.7 | 0.845631 | **-26±10.6** | **0.014249** | **33.7±10.5** | **0.001427** | -10.6±10.6 | 0.317865 | **0.4** |
| EAACMCTC178-SATT578 | SAT_222-EACAMCTT122 | -1.3±4.3 | 0.763014 | -2.9±4.2 | 0.497582 | 2.2±4.2 | 0.610683 | 2.0±4.2 | 0.635759 | 0.1 |
| SATT184-SAT_353 | GMLPSI2-SCT_189 | -10.7±7.6 | 0.154832 | 13.7±7.5 | 0.067142 | -13.1±7.4 | 0.078889 | 10.2±7.4 | 0.166270 | 0.3 |
| EACAMCTT123-BE475343 | SATT346-SAT_147 | **26.6±9.3** | **0.004397** | -16.1±9.3 | 0.082102 | **-30.5±9.2** | **0.000944** | **19.4±9.1** | **0.033904** | **0.6** |
| SATT634-SATT266 | EAACMCAC175-SATT244 | 0.0±0.1 | 0.999884 | 0.0±0.1 | 0.996626 | 0.0±0.1 | 0.997810 | 0.0±0.1 | 0.998810 | 0.0 |
| SATT634-SATT266 | EAACMCTC276-SCTT011 | 0.0±0.1 | 0.998266 | 0.0±0.1 | 0.997238 | 0.0±0.1 | 0.998973 | 0.0±0.1 | 0.999943 | 0.0 |
| SCT_189-SATT440 | SATT435-SATT323 | 0.0±0.1 | 0.998819 | 0.0±0.1 | 0.998094 | 0.0±0.1 | 0.997231 | 0.0±0.1 | 0.997843 | 0.0 |
| *Daidzein* |  |  |  |  |  |  |  |  |  |  |
| SATT174-SATT236 | SATT187-EAGGMCTT205 | **12.4±5.8** | **0.034559** | 6.4±5.8 | 0.271685 | **-13.5±5.8** | **0.019341** | **-5.3±5.7** | 0.358968 | **0.2** |
| SATT187-EAGGMCTT205 | SATT175-EAGGMCTT095 | 1.4±2.7 | 0.606938 | -1.2±2.7 | 0.641683 | 0.1±2.7 | 0.959516 | -0.2±2.7 | 0.928118 | 0.1 |
| SATT396-SAT_337 | SAT_339-SATT280 | 0.0±0.1 | 0.998977 | 0.0±0.1 | 0.998383 | 0.0±0.1 | 0.999419 | 0.0±0.1 | 0.999968 | 0.0 |
| SAT_106-AW781285 | SATT175-EAGGMCTT095 | -12.7±6.6 | 0.054521 | -2.4±6.5 | 0.715735 | 7.5±6.5 | 0.248558 | 7.6±6.5 | 0.240601 | 0.1 |
| SATT591-AZ536570 | SAT_279-EACAMCTT123 | 1.9±3.7 | 0.600516 | -4.0±3.7 | 0.276883 | 1.9±3.7 | 0.598402 | 0.1±3.7 | 0.974837 | 0.1 |
| SAT_272-SAT_411 | SATT674-SAT_228 | 4.1±5.0 | 0.411107 | 5.3±4.9 | 0.281838 | -5.7±4.9 | 0.248591 | -3.6±4.9 | 0.465751 | 0.2 |
| SATT359-EACAMCTT067 | SATT309-RHG1_INDEL | -5.5±6.3 | 0.379508 | 2.1±6.2 | 0.727818 | **12.6±6.2** | **0.040912** | -9.2±6.1 | 0.132754 | **0.1** |
| SAT_342-EAACMCTC384 | SAT_380-SATT263 | **-12.5±6.3** | **0.048070** | -8.5±6.3 | 0.175356 | 8.3±6.2 | 0.183444 | **12.7±6.2** | **0.041402** | **0.2** |
| SAT_062-SATT281 | EAACMCAC175-SATT244 | 10.1±5.8 | 0.081288 | 1.8±5.7 | 0.745338 | -0.7±5.7 | 0.900683 | **-11.2±5.7** | **0.048627** | **0.2** |
| EAACMCTC063-EACAMCTT419 | SATT302-SATT142 | 0.0±0.1 | 0.995264 | 0.0±0.1 | 0.999015 | 0.0±0.1 | 0.996995 | 0.0±0.1 | 0.999329 | 0.0 |
| EAACMCTC063-EACAMCTT419 | SATT522-AW756935 | 1.7±3.0 | 0.573955 | 0.3±3.0 | 0.914130 | -1.9±3.0 | 0.521133 | -0.1±3.0 | 0.981270 | 0.0 |
| SATT266-SAT_135 | SAT_282-SAT_242 | 0.0±0.1 | 0.992825 | 0.0±0.1 | 0.995894 | 0.0±0.1 | 0.998995 | 0.0±0.1 | 0.997960 | 0.4 |
| SAT_333-SCT_192 | SAT_284-SATT372 | 0.0±0.1 | 0.996223 | 0.0±0.1 | 0.996056 | 0.0±0.1 | 0.999983 | 0.0±0.1 | 0.992294 | 0.0 |
| EACAMCTT122-SATT082 | SATT167-SATT552 | 0.0±0.1 | 0.995527 | 0.0±0.1 | 0.996210 | 0.0±0.1 | 0.989970 | 0.0±0.1 | 0.998041 | 0.1 |
| SATT691-SATT598 | SAT_190-SCAA001 | 0.0±0.1 | 0.999257 | 0.0±0.1 | 0.996321 | 0.0±0.1 | 0.998033 | 0.0±0.1 | 0.995003 | 0.0 |
| SAT_380-SATT263 | EAGGMCTT098-SATT009 | -6.2±5.4 | 0.252001 | 0.6±5.4 | 0.916626 | -3.8±5.4 | 0.476564 | 9.5±5.3 | 0.074954 | 0.1 |
| SAT_380-SATT263 | SATT490-SAT_197 | 0.0±0.1 | 0.998028 | 0.0±0.1 | 0.998928 | 0.0±0.1 | 0.997041 | 0.0±0.1 | 0.998112 | 0.1 |
| EAGGMCAG365-EAGGMCAG422 | SATT522-AW756935 | 0.8±4.5 | 0.861267 | 5.9±4.5 | 0.187445 | -6.6±4.4 | 0.135389 | -0.1±4.4 | 0.984113 | 0.2 |
| SATT242-EAACMCAC227 | SATT339-SATT257 | 9.8±5.7 | 0.088408 | 3.9±5.7 | 0.488151 | -4.6±5.7 | 0.411763 | -9.1±5.6 | 0.106045 | 0.2 |
| EAGGMCTT176-EAGGMCT135 | SATT683-SAT_275 | 6.3±5.7 | 0.267811 | -0.9±5.7 | 0.875932 | -6.7±5.7 | 0.238132 | 1.2±5.6 | 0.830709 | 0.1 |
| SAT_121-SATT346 | SATT259-SATT188 | -3.5±7.5 | 0.646135 | -7.0±7.4 | 0.347878 | -13.4±7.4 | 0.068835 | **23.9±7.4** | **0.001270** | **0.3** |
| *Glycitein* |  |  |  |  |  |  |  |  |  |  |
| SATT385-SATT619 | SAT_389-SATT636 | 0.0±0.0 | 0.997596 | 0.0±0.0 | 0.987060 | 0.0±0.0 | 0.998840 | 0.0±0.0 | 0.985849 | 0.2 |
| EAACMCAC086-SATT187 | SAT_247-SATT519 | 0.0±0.0 | 0.997280 | 0.0±0.0 | 0.998274 | 0.0±0.0 | 0.991030 | 0.0±0.0 | 0.986513 | 0.1 |
| SATT304-SATT416 | EACAMCTT122-SATT082 | **5.6±2** | **0.004862** | **-8.1±1.9** | **0.000031** | 2.7±1.9 | 0.153073 | -0.3±1.9 | 0.856714 | **0.6** |
| EAACMCTC178-SATT578 | EAACMCTC379-SATT316 | 0.4±1.5 | 0.819212 | 1.1±1.5 | 0.464833 | 1.7±1.5 | 0.264634 | **-3.2±1.5** | **0.030736** | **0.2** |
| SAT_135-SATT546 | SAT_284-SATT372 | -1.4±1.9 | 0.456174 | **-4.6±1.9** | **0.015952** | 1.9±1.9 | 0.322396 | **4.2±1.9** | **0.026453** | **0.3** |
| SATT458-SAT_284 | SAT_086-SATT256 | 0.0±0.0 | 0.988014 | 0.0±0.0 | 0.999154 | 0.0±0.0 | 0.994045 | 0.0±0.0 | 0.992959 | 0.1 |
| SATT269-SATT160 | EAGGMCTT164-SATT330 | **8.5±1.9** | **0.000020** | 3.5±1.9 | 0.066585 | **-7.6±1.9** | **0.000069** | **-4.3±1.9** | **0.022641** | **0.8** |
| *Total isoflavones* |  |  |  |  |  |  |  |  |  |  |
| SAT_279-EACAMCTT123 | SAT_112-SATT691 | 0.0±0.1 | 0.997080 | 0.0±0.1 | 0.998358 | 0.0±0.1 | 0.996427 | 0.0±0.1 | 0.997729 | 0.1 |
| SAT_265-EAAGMCAT279 | SAT_279-EACAMCTT123 | 9.9±11.2 | 0.375627 | -7.9±11.2 | 0.477855 | 0.3±11.1 | 0.979267 | -2.4±11.2 | 0.830226 | 0.1 |
| SATT089-SATT233 | SAT_275-SATT387 | 5.0±17.2 | 0.773220 | 31.2±17.0 | 0.066297 | -2.5±16.9 | 0.885125 | **-33.7±16.9** | **0.046146** | **0.2** |
| SATT455-SATT228 | SATT304-SATT416 | 0.0±0.1 | 0.998614 | 0.0±0.1 | 0.997278 | 0.0±0.1 | 0.996404 | 0.0±0.1 | 0.997841 | 0.1 |
| SATT281-SATT291 | AW756935-SAT_090 | 12.3±11.4 | 0.280744 | -16.9±11.3 | 0.134341 | 2.2±11.3 | 0.843100 | 2.5±11.2 | 0.825401 | 0.2 |
| SCT_033-SCT_188 | SATT683-SAT_275 | 0.0±0.1 | 0.999414 | 0.0±0.1 | 0.999403 | 0.0±0.1 | 0.998899 | 0.0±0.1 | 0.998831 | 0.0 |
| SATT292-SAT_419 | EAACMCTC276-SCTT011 | **-46.7±19.7** | **0.017936** | -6.1±19.7 | 0.757187 | 25.5±19.4 | 0.188883 | 27.0±19.4 | 0.163522 | **0.3** |
| SCT_189-SATT440 | SCT_195-SATT159 | -11.1±6.9 | 0.877504 | -4.0±6.9 | 0.562863 | 5.0±6.9 | 0.468308 | 0.0±6.9 | 0.996057 | 0.1 |
| ^a^Marker interval within each epistatic interaction occurs. ^b^AAE1, AAE2, AAE3, and AAE4 are the additive-additive by environment interaction effect in μg/g plus/minus standard error for BREC_06 (AE1), DRC_06 (AE2), BREC_07 (AE3), and DRC_07 (AE4). P-values represent the significance of each effect. ^c^Heritability of the additive-additive by environment interaction effect (%). P-values represent the significance of each effect. | | | | | | | | | | |
