# Supplementary material for: Intricate environment-modulated genetic networks control isoflavone accumulation in soybean seeds
Source: BMC Plant Biol. 2010 Jun 11;10:105. doi: 10.1186/1471-2229-10-105 (PMC3224685; doi:10.1186/1471-2229-10-105)
Supplement: Additional file 6 — Neighbor-joining phyllogenetic tree for chalcone synthase protein entries. Neighbor-joining phyllogenetic tree generated using AlignX (Invitrogen) using NCBI soybean chalcone synthase protein entries. [file 1471-2229-10-105-S6.DOC]

**Additional File 6**. Neighbor-joining phyllogenetic tree generated using AlignX (Invitrogen) using NCBI soybean chalcone synthase protein entries (NCBI entries: CHS1 ABB30178; CHS2 CAA46590; CHS3 X53958; CHS4 CAA36317; CHS5 AAB01004; CHS6 AAA33951; CHS7 AAA33950; CHS8 AAO67373; CHS9 ABQ63059) and the two putative CHS6 predicted proteins present in Glyma1.01 (Glyma01g22880.1 and Glyma09g08780.1).
