# Supplementary material for: Intricate environment-modulated genetic networks control isoflavone accumulation in soybean seeds
Source: BMC Plant Biol. 2010 Jun 11;10:105. doi: 10.1186/1471-2229-10-105 (PMC3224685; doi:10.1186/1471-2229-10-105)
Supplement: Additional file 7 — Neighbor-joining phyllogenetic tree for chalcone synthase coding sequence entries. Neighbor-joining phyllogenetic tree generated using AlignX (Invitrogen) using NCBI soybean chalcone synthase coding sequence entries coding sequence. [file 1471-2229-10-105-S7.DOC]

**Additional File 7**. Neighbor-joining phyllogenetic tree generated using AlignX (Invitrogen) using NCBI soybean chalcone synthase coding sequence entries coding sequence (NCBI entries: CHS1 DQ239918; CHS2 X65636; CHs3 X53958; CHS4 X52097; CHS5 L07647; CHS6 L03352; CHS7 M98871; CHS8 AY237728; CHS9 EF623853) and two putative CHS6 predicted coding sequences present in Glyma1.01 (Glyma01g22880.1 and Glyma09g08780.1).
