# Supplementary material for: Intricate environment-modulated genetic networks control isoflavone accumulation in soybean seeds
Source: BMC Plant Biol. 2010 Jun 11;10:105. doi: 10.1186/1471-2229-10-105 (PMC3224685; doi:10.1186/1471-2229-10-105)
Supplement: Additional file 8 — Protein alignment of chalcone synthase. Protein alignment of NCBI soybean chalcone synthase VI protein sequence and the two putative CHS6 predicted predicted proteins present in Glyma1.01 [file 1471-2229-10-105-S8.DOC]

1 50

AAA33951 SOYCHSVI (1) MVSVEEIRKAQRAEGPATVMAIGTATPPNCVDQSTYPDYYFRITNSDHMN

Glyma01g22880.1 (1) MVSVEEIRKAQRAEGPATVMAIGTATPPNCVDQSTYPDYYFRITNSDHMT

Glyma09g08780.1 (1) MVSVEEIRKAQRAEGPATVMAIGTATPPNCVDQSTYPDYYFRITNSDHMN

51 100

AAA33951 SOYCHSVI (51) ELKEKFKRMCDKSMIKKRYMYLNEEILKENPSVCAYMEPSLDARQDMVVV

Glyma01g22880.1 (51) ELKEKFKRMCDKSMIKKRYMYLNEEILKENPSVCAYMAPSLDARQDMVVV

Glyma09g08780.1 (51) ELKEKFKRMCDKSMIKKRYMYLNEEILKENPSVCAYMEPSLDARQDMVVV

101 150

AAA33951 SOYCHSVI (101) EVPKLGKEAATKAIKEWGQPKSKITHLIFCTTSGVDMPGADYQLTKLLGL

Glyma01g22880.1 (101) EVPKLGKEAATKAIKEWGQPKSKITHLIFCTTSGVDMPGADYQLTKLLGL

Glyma09g08780.1 (101) EVPKLGKEAATKAIKEWGQPKSKITHLIFCTTSGVDMPGADYQLTKLLGL

151 200

AAA33951 SOYCHSVI (151) RPSVKRYMMYQQGCFAGGTVLRLAKDLAENNTGARVLVVCSEITAVTFRG

Glyma01g22880.1 (151) RPSVKRYMMYQQGCFAGGTVLRLAKDLAENNTGARVLVVCSEITAVTFRG

Glyma09g08780.1 (151) RPSVKRYMMYQQGCFAGGTVLRLAKDLAENNTGARVLVVCSEITAVTFRG

201 250

AAA33951 SOYCHSVI (201) PSDTHLDSLVGQALFGDGAAAVIVGSDPLPAEKPLFELVWTAQTILPDSE

Glyma01g22880.1 (201) PSDTHLDSLVGQALFGDGAAAVILGSDPLPAEKPLFELVWTAQTILPDSE

Glyma09g08780.1 (201) PSDTHLDSLVGQALFGDGAAAVIVGSDPLPAEKPLFELVWTAQTILPDSE

251 300

AAA33951 SOYCHSVI (251) GAIDGHLREVGLTFHLLKDVPGLISKNIQKALVEAFQPLGIDDYNSIFWI

Glyma01g22880.1 (251) GAIDGHLREVGLTFHLLKDVPGLISKNIQKALVEAFQPLGIDDYNSIFWI

Glyma09g08780.1 (251) GAIDGHLREVGLTFHLLKDVPGLISKNIQKALVEAFQPLGIDDYNSIFWI

301 350

AAA33951 SOYCHSVI (301) AHPGGPAILDQVEAKLGLKPEKMEATRHVLSEYGNMSSACVLFILDQMRK

Glyma01g22880.1 (301) AHPGGPAILDQVEAKLGLKPEKMEATRHVLSEYGNMSSACVLFILDQMRK

Glyma09g08780.1 (301) AHPGGPAILDQVEAKLGLKPEKMEATRHVLSEYGNMSSACVLFILDQMRK

351 389

AAA33951 SOYCHSVI (351) KSIENGLGTTGEGLEWGVLFGFGPGLTVETVVLRSVTV-

Glyma01g22880.1 (351) KSIENGLGTTGEGLEWGVLFGFGPGLTVETVVLRSVTV-

Glyma09g08780.1 (351) KSIENGLGTTGEGLEWGVLFGFGPGLTVETVVLRSVTV-

**Additional File 8**. Protein alignment of NCBI soybean chalcone synthase VI protein sequence and the two putative CHS6 predicted predicted proteins present in Glyma1.01 (Glyma01g22880.1 and Glyma09g08780.1).
