# Supplementary material for: Intricate environment-modulated genetic networks control isoflavone accumulation in soybean seeds
Source: BMC Plant Biol. 2010 Jun 11;10:105. doi: 10.1186/1471-2229-10-105 (PMC3224685; doi:10.1186/1471-2229-10-105)
Supplement: Additional file 9 — Alignment of chalcone synthase coding region sequences. Alignment of NCBI soybean chalcone synthase VI coding region sequence and the two putative CHS6 predicted coding regions present in Glyma1.01 [file 1471-2229-10-105-S9.DOC]

1 50

L03352 CHS6cds (1) ATGGTGAGTGTTGAAGAGATTCGTAAGGCACAACGTGCAGAAGGCCCTGC

Glyma01g22880.1:cds (1) ATGGTGAGTGTTGAAGAGATTCGTAAGGCACAACGTGCAGAAGGCCCTGC

Glyma09g08780.1:cds (1) ATGGTGAGTGTTGAAGAGATTCGTAAGGCACAACGTGCAGAAGGCCCTGC

51 100

L03352 CHS6cds (51) CACCGTGATGGCTATTGGCACGGCCACTCCTCCCAACTGCGTGGATCAGA

Glyma01g22880.1:cds (51) CACCGTGATGGCTATTGGCACGGCCACTCCTCCCAACTGCGTGGATCAGA

Glyma09g08780.1:cds (51) CACCGTGATGGCTATTGGCACGGCCACTCCTCCCAACTGCGTGGATCAGA

101 150

L03352 CHS6cds (101) GTACCTATCCTGACTATTATTTCCGCATCACCAACAGTGACCACATGAAC

Glyma01g22880.1:cds (101) GTACCTATCCTGACTATTATTTCCGCATCACCAACAGTGACCACATGACC

Glyma09g08780.1:cds (101) GTACCTATCCTGACTATTATTTCCGCATCACCAACAGTGACCACATGAAC

151 200

L03352 CHS6cds (151) GAGCTCAAAGAAAAGTTCAAGCGCATGTGTGATAAGTCAATGATTAAGAA

Glyma01g22880.1:cds (151) GAGCTCAAAGAAAAGTTCAAGCGCATGTGTGATAAGTCAATGATTAAGAA

Glyma09g08780.1:cds (151) GAGCTCAAAGAAAAGTTCAAGCGCATGTGTGATAAGTCAATGATTAAGAA

201 250

L03352 CHS6cds (201) ACGATACATGTACTTAAATGAAGAGATCCTGAAGGAGAATCCAAGTGTTT

Glyma01g22880.1:cds (201) ACGATACATGTACTTAAATGAAGAGATCCTGAAGGAGAATCCGAGTGTTT

Glyma09g08780.1:cds (201) ACGATACATGTACTTAAATGAAGAGATCCTGAAGGAGAATCCAAGTGTTT

251 300

L03352 CHS6cds (251) GTGCCTATATGGAACCTTCATTGGATGCAAGGCAAGACATGGTGGTTGTG

Glyma01g22880.1:cds (251) GTGCCTATATGGCACCTTCATTGGATGCAAGGCAAGACATGGTGGTTGTG

Glyma09g08780.1:cds (251) GTGCCTATATGGAACCTTCATTGGATGCAAGGCAAGACATGGTGGTTGTG

301 350

L03352 CHS6cds (301) GAGGTACCAAAGTTGGGAAAAGAGGCTGCAACAAAGGCAATCAAGGAATG

Glyma01g22880.1:cds (301) GAGGTACCAAAGTTGGGAAAAGAGGCTGCAACAAAGGCAATCAAGGAATG

Glyma09g08780.1:cds (301) GAGGTACCAAAGTTGGGAAAAGAGGCTGCAACAAAGGCAATCAAGGAATG

351 400

L03352 CHS6cds (351) GGGTCAACCCAAGTCCAAGATTACTCATCTCATCTTCTGCACCACTAGTG

Glyma01g22880.1:cds (351) GGGTCAACCCAAGTCCAAGATTACTCATCTCATCTTCTGCACCACTAGTG

Glyma09g08780.1:cds (351) GGGTCAACCCAAGTCCAAGATTACTCATCTCATCTTCTGCACCACTAGTG

401 450

L03352 CHS6cds (401) GTGTGGACATGCCTGGTGCTGATTATCAGCTCACAAAACTGCTAGGACTT

Glyma01g22880.1:cds (401) GTGTGGACATGCCTGGTGCTGATTATCAGCTCACAAAACTGCTAGGACTT

Glyma09g08780.1:cds (401) GTGTGGACATGCCTGGTGCTGATTATCAGCTCACAAAACTGCTAGGACTT

451 500

L03352 CHS6cds (451) CGTCCCTCCGTCAAGCGTTACATGATGTACCAACAAGGCTGCTTTGCTGG

Glyma01g22880.1:cds (451) CGTCCCTCCGTCAAGCGTTACATGATGTACCAACAAGGCTGCTTTGCTGG

Glyma09g08780.1:cds (451) CGTCCCTCCGTCAAGCGTTACATGATGTACCAACAAGGCTGCTTTGCTGG

501 550

L03352 CHS6cds (501) TGGCACGGTGCTTCGTCTGGCGAAAGACTTGGCCGAAAACAACACGGGTG

Glyma01g22880.1:cds (501) TGGCACGGTGCTTCGTCTGGCGAAAGACTTGGCCGAAAACAACACGGGTG

Glyma09g08780.1:cds (501) TGGCACGGTGCTTCGTCTGGCGAAAGACTTGGCCGAAAACAACACGGGTG

551 600

L03352 CHS6cds (551) CTCGTGTGCTCGTCGTGTGTTCAGAGATCACAGCAGTCACATTTCGCGGC

Glyma01g22880.1:cds (551) CTCGTGTGCTCGTCGTGTGTTCAGAGATCACAGCAGTCACATTTCGCGGC

Glyma09g08780.1:cds (551) CTCGTGTGCTCGTCGTGTGTTCAGAGATCACAGCAGTCACATTTCGCGGC

601 650

L03352 CHS6cds (601) CCGAGTGACACCCATCTTGATAGCCTTGTTGGGCAAGCCTTGTTTGGAGA

Glyma01g22880.1:cds (601) CCGAGTGACACCCATCTTGATAGCCTTGTTGGGCAAGCCTTGTTTGGAGA

Glyma09g08780.1:cds (601) CCGAGTGACACCCATCTTGATAGCCTTGTTGGGCAAGCCTTGTTTGGAGA

651 700

L03352 CHS6cds (651) TGGTGCAGCTGCTGTCATTGTTGGATCAGACCCTTTGCCTGCTGAAAAGC

Glyma01g22880.1:cds (651) TGGTGCAGCTGCTGTCATTCTTGGATCAGACCCTTTGCCTGCTGAAAAGC

Glyma09g08780.1:cds (651) TGGTGCAGCTGCTGTCATTGTTGGATCAGACCCTTTGCCTGCTGAAAAGC

701 750

L03352 CHS6cds (701) CTTTGTTTGAGCTTGTGTGGACTGCACAAACAATCCTGCCAGACAGTGAA

Glyma01g22880.1:cds (701) CTTTGTTTGAGCTTGTGTGGACTGCACAAACAATCCTTCCAGACAGTGAA

Glyma09g08780.1:cds (701) CTTTGTTTGAGCTTGTGTGGACTGCACAAACAATCCTGCCAGACAGTGAA

751 800

L03352 CHS6cds (751) GGGGCTATTGATGGCCACCTTCGCGAAGTAGGACTCACTTTCCATCTCCT

Glyma01g22880.1:cds (751) GGGGCTATTGATGGCCACCTTCGCGAAGTAGGACTCACTTTCCATCTCCT

Glyma09g08780.1:cds (751) GGGGCTATTGATGGCCACCTTCGCGAAGTAGGACTCACTTTCCATCTCCT

801 850

L03352 CHS6cds (801) CAAGGATGTTCCTGGACTCATCTCGAAGAACATCCAAAAGGCCTTGGTTG

Glyma01g22880.1:cds (801) CAAGGATGTTCCTGGACTCATCTCGAAGAACATCCAAAAGGCCTTGGTTG

Glyma09g08780.1:cds (801) CAAGGATGTTCCTGGACTCATCTCGAAGAACATCCAAAAGGCCTTGGTTG

851 900

L03352 CHS6cds (851) AAGCCTTCCAACCCTTGGGAATTGATGATTACAACTCTATCTTTTGGATT

Glyma01g22880.1:cds (851) AAGCCTTCCAACCCTTGGGAATTGATGATTACAACTCTATCTTTTGGATT

Glyma09g08780.1:cds (851) AAGCCTTCCAACCCTTGGGAATTGATGATTACAACTCTATCTTTTGGATT

901 950

L03352 CHS6cds (901) GCACACCCTGGTGGACCAGCAATATTGGACCAAGTTGAGGCTAAGTTAGG

Glyma01g22880.1:cds (901) GCACACCCTGGTGGACCAGCAATATTGGACCAAGTTGAGGCTAAGTTAGG

Glyma09g08780.1:cds (901) GCACACCCTGGTGGACCAGCAATATTGGACCAAGTTGAGGCTAAGTTAGG

951 1000

L03352 CHS6cds (951) CTTGAAACCTGAAAAAATGGAAGCTACTAGACATGTGCTCAGCGAGTATG

Glyma01g22880.1:cds (951) CTTGAAACCTGAAAAAATGGAAGCTACTAGACATGTGCTCAGCGAGTATG

Glyma09g08780.1:cds (951) CTTGAAACCTGAAAAAATGGAAGCTACTAGACATGTGCTCAGCGAGTATG

1001 1050

L03352 CHS6cds (1001) GTAACATGTCAAGTGCATGCGTGTTGTTCATCTTGGATCAAATGAGGAAG

Glyma01g22880.1:cds (1001) GTAACATGTCAAGTGCATGCGTGTTGTTCATCTTGGATCAAATGAGGAAG

Glyma09g08780.1:cds (1001) GTAACATGTCAAGTGCATGCGTGTTGTTCATCTTGGATCAAATGAGGAAG

1051 1100

L03352 CHS6cds (1051) AAGTCAATAGAAAATGGACTTGGCACCACTGGTGAAGGGCTTGAATGGGG

Glyma01g22880.1:cds (1051) AAGTCAATAGAAAATGGACTTGGCACCACTGGTGAAGGGCTTGAATGGGG

Glyma09g08780.1:cds (1051) AAGTCAATAGAAAATGGACTTGGCACCACTGGTGAAGGGCTTGAATGGGG

1101 1150

L03352 CHS6cds (1101) TGTGCTATTTGGTTTTGGCCCTGGACTCACTGTTGAGACTGTTGTGCTTC

Glyma01g22880.1:cds (1101) TGTGCTATTTGGTTTTGGCCCTGGACTCACTGTTGAGACTGTTGTGCTTC

Glyma09g08780.1:cds (1101) TGTGCTATTTGGTTTTGGCCCTGGACTCACTGTTGAGACTGTTGTGCTTC

1151 1167

L03352 CHS6cds (1151) GCAGTGTCACAGTCTAA

Glyma01g22880.1:cds (1151) GCAGTGTCACAGTCTAA

Glyma09g08780.1:cds (1151) GCAGTGTCACAGTCTAA

**Additional File 9**. Alignment of NCBI soybean chalcone synthase VI coding region sequence and the two putative CHS6 predicted coding regions present in Glyma1.01 (Glyma01g22880.1 and Glyma09g08780.1).
