# Supplementary material for: Intricate environment-modulated genetic networks control isoflavone accumulation in soybean seeds
Source: BMC Plant Biol. 2010 Jun 11;10:105. doi: 10.1186/1471-2229-10-105 (PMC3224685; doi:10.1186/1471-2229-10-105)
Supplement: Additional file 10 — Monte Carlo simulation results. Summarized Monte Carlo simulation results for mapping QTLs with A and AxE effects. [file 1471-2229-10-105-S10.doc]

| Additional file 10. Summarized Monte Carlo simulation results for mapping QTLs with A and AxE effects. | | | | | | | | | | | | | | | | | | |
| --- | --- | --- | --- | --- | --- | --- | --- | --- | --- | --- | --- | --- | --- | --- | --- | --- | --- | --- |
| QTL | Chr. | ^a^RC (A) | ^a^RC (AE) | ^a^RC | Pos. (cM) | Est.(SE) | ^b^SI (cM) | A | Est.(SE) | AE1 | Est.(SE) | AE2 | Est.(SE) | AE3 | Est.(SE) | AE4 | Est.(SE) | Power |
| Q1 | Gm08 | 0.3184 | 0.042 | 0.3604 | 37.45 | 37.98(1.39) | 2.9 | -12.0 | -11.52(0.82) | -4.7 | -4.34(0.47) | -2.6 | -2.41(0.35) | 2.5 | 2.33(0.37) | 4.7 | 4.42(0.49) | 100 |
| Q2 | Gm11 | 0.1415 | 0.0265 | 0.168 | 97.03 | 96.70(1.44) | 4.8 | 8.0 | 8.37(0.83) | -1.3 | -1.25(0.36) | -1.2 | -1.26(0.26) | -2.6 | -2.55(0.31) | 5.1 | 5.16(0.43) | 100 |
| Q3 | Gm14 | 0.0796 | 0.0265 | 0.1061 | 47.06 | 46.85(1.53) | 3.4 | 6.0 | 5.69(0.71) | 3.3 | 3.02(0.45) | 2.3 | 2.05(0.37) | -1.3 | -1.20(0.30) | -4.3 | -3.87(0.57) | 100 |
| Q4 | Gm04 | 0.0553 | 0 | 0.0553 | 48.96 | 48.95(1.26) | 2.6 | 5.0 | 4.96(0.69) | 0.0 | 0.00(0.00) | 0.0 | 0.00(0.00) | 0.0 | -0.00(0.01) | 0.0 | 0.00(0.00) | 100 |
| Q5 | Gm02 | 0.1083 | 0.0155 | 0.1238 | 57.04 | 57.64(2.00) | 5 | -7.0 | -6.44(1.05) | -2.6 | -2.10(0.64) | 0.8 | 0.66(0.35) | 3.3 | 2.78(0.72) | -1.6 | -1.34(0.43) | 97 |
| Q6 | Gm15 | 0.0553 | 0.0287 | 0.084 | 13.3 | 13.32(0.70) | 2.5 | -5.0 | -4.86(0.63) | 4.0 | 3.76(0.38) | -0.5 | -0.53(0.25) | 1.2 | 1.15(0.31) | -4.6 | -4.36(0.47) | 100 |
| Q7 | Gm18 | 0 | 0.0398 | 0.0398 | 47.38 | 47.26(0.65) | 3.5 | 0.0 | 0.04(0.62) | 3.3 | 3.18(0.35) | 0.3 | 0.21(0.25) | -6.1 | -5.92(0.33) | 2.6 | 2.45(0.37) | 100 |
| Q8 | Gm20 | 0.0271 | 0.0111 | 0.0382 | 14.16 | 14.45(1.74) | 4.1 | -3.5 | -3.52(0.69) | -3.3 | -3.12(0.47) | 0.8 | 0.68(0.26) | 1.5 | 1.41(0.32) | 1.1 | 1.01(0.33) | 100 |
| Q9 | Gm19 | 0.0088 | 0.0066 | 0.0154 | 14.31 | 14.97(4.19) | 11.5 | 2.0 | 1.93(0.66) | 1.6 | 1.24(0.47) | -2.4 | -1.77(0.77) | 0.1 | 0.00(0.27) | 0.7 | 0.53(0.40) | 68 |
| Q10 | Gm07 | 0.0088 | 0 | 0.0088 | 88.37 | 87.79(4.44) | 7.8 | -2.0 | -2.18(0.53) | 0.0 | -0.00(0.00) | 0.0 | -0.00(0.00) | 0.0 | 0.00(0.00) | 0.0 | 0.00(0.00) | 58 |
| ^a^RC relative contribution of each additive (A) and additive by environment (AE) effect. ^b^SI, average length of support interval of 100 simulation replicates. Each estimate represents the average of the estimates from 100 runs with a standard error of SE. The estimated false discovery rate of QTL was 0.1875 | | | | | | | | | | | | | | | | | | |
