# Supplementary material for: Intricate environment-modulated genetic networks control isoflavone accumulation in soybean seeds
Source: BMC Plant Biol. 2010 Jun 11;10:105. doi: 10.1186/1471-2229-10-105 (PMC3224685; doi:10.1186/1471-2229-10-105)
Supplement: Additional file 11 — NCBI entries used for candidate gene identification. [file 1471-2229-10-105-S11.doc]

**Additional File 11: NCBI entries used for candidate gene identification**

X52953

Glycine max PAL1 gene for phenylalanine ammonia lyase (EC 4.3.1.5)

gi|18376|emb|X52953.1|[18376]

X55730

Soybean mRNA for reductase involved in deoxychalcone synthesis (NAD(P)H dependent 6'-deoxychalcone synthase)

gi|18727|emb|X55730.1|[18727]

X92437

Glycine max mRNA for cinnamic acid 4-hydroxylase (CYP73)

gi|1044867|emb|X92437.1|[1044867]

AF167556

Glycine max dihydroflavonol-4-reductase DFR1 mRNA, complete cds

gi|5852932|gb|AF167556.1|[5852932]

AF202183

Glycine max isoflavone reductase homolog 1 (IFR1) mRNA, complete cds

gi|6573168|gb|AF202183.1|[6573168]

AF202184

Glycine max isoflavone reductase homolog 2 (IFR2) mRNA, complete cds

gi|6573170|gb|AF202184.1|[6573170]

AF195798

Glycine max isoflavone synthase 1 (ifs1) mRNA, complete cds

gi|6979519|gb|AF195798.1|[6979519]

AF195799

Glycine max isoflavone synthase 2 (ifs2) mRNA, complete cds

gi|6979521|gb|AF195799.1|[6979521]

X69955

Glycine max mRNA for 4-coumarate:CoA ligase (4CL4 gene)

gi|13559168|emb|X69955.2|[13559168]

AF279267

Glycine max 4-coumarate:coenzyme A ligase (4CL1) mRNA, complete cds

gi|19773581|gb|AF279267.1|[19773581]

AY595413

Glycine max chalcone isomerase 1A mRNA, complete cds

gi|51039621|gb|AY595413.1|[51039621]

AY595414

Glycine max chalcone isomerase 1B1 mRNA, complete cds

gi|51039623|gb|AY595414.1|[51039623]

AY595415

Glycine max chalcone isomerase 2 mRNA, complete cds

gi|51039625|gb|AY595415.1|[51039625]

AY595416

Glycine max putative chalcone isomerase 3 mRNA, complete cds

gi|51039627|gb|AY595416.1|[51039627]

AY595417

Glycine max putative chalcone isomerase 4 mRNA, complete cds

gi|51039629|gb|AY595417.1|[51039629]

AY595420

Glycine max flavanone 3-hydroxylase mRNA, complete cds

gi|51039636|gb|AY595420.1|[51039636]

AY669326

Glycine max flavanone 3-hydroxylase (F3H2) gene, complete cds

gi|51556894|gb|AY669326.1|[51556894]

AB154415

Glycine max HIDH mRNA for 2-hydroxyisoflavanone dehydratase, complete cds

gi|56692179|dbj|AB154415.1|[56692179]

AY942159

Medicago truncatula SAM dependent isoflavone 7-O-methyltransferase mRNA, complete cds

gi|62871344|gb|AY942159.1|[62871344]

DQ026299

Glycine max dihydroflavonol-4-reductase (DFR2) mRNA, partial cds

gi|66356299|gb|DQ026299.1|[66356299]

DQ191403

Glycine max cultivar L66-14 chalcone isomerase 3 (CHI3) gene, complete cds

gi|77456096|gb|DQ191403.1|[77456096]

EU921437

Glycine max chalcone reductase mRNA, complete cds

gi|197091509|gb|EU921437.1|[197091509]

EU925557

Glycine max vestitone reductase mRNA, complete cds

gi|197215942|gb|EU925557.1|[197215942]
